# Perceptions of diabetes risk and prevention in Nairobi, Kenya: A qualitative and theory of change development study

**DOI:** 10.1371/journal.pone.0297779

**Published:** 2024-02-13

**Authors:** Anthony Muchai Manyara, Elizabeth Mwaniki, Jason M. R. Gill, Cindy M. Gray

**Affiliations:** 1 School of Health and Wellbeing, University of Glasgow, Glasgow, United Kingdom; 2 Department of Health Systems Management and Public Health, Technical University of Kenya, Nairobi, Kenya; 3 Global Health and Ageing Research Unit, University of Bristol, Bristol, United Kingdom; 4 School of Cardiovascular and Metabolic Health, University of Glasgow, Glasgow, United Kingdom; 5 School of Social and Political Sciences, University of Glasgow, Glasgow, United Kingdom; Department of Medicine, University of Cape Town, SOUTH AFRICA

## Abstract

**Background:**

Type 2 diabetes is increasing in Kenya, especially in urban settings, and prevention interventions based on local evidence and context are urgently needed. Therefore, this study aimed to explore diabetes risk and co-create a diabetes prevention theory of change in two socioeconomically distinct communities to inform future diabetes prevention interventions.

**Methods:**

In-depth interviews were conducted with middle-aged residents in two communities in Nairobi (one low-income (n = 15), one middle-income (n = 14)), and thematically analysed. The theory of change for diabetes prevention was informed by analysis of the in-depth interviews and the Behaviour Change Wheel framework, and reviewed by a sub-set (n = 13) of interviewees.

**Results:**

The key factors that influenced diabetes preventive practices in both communities included knowledge and skills for diabetes prevention, understanding of the benefits/consequences of (un)healthy lifestyle, social influences (e.g., upbringing, societal perceptions), and environmental contexts (e.g., access to (un)healthy foods and physical activity facilities). The proposed strategies for diabetes prevention included: increasing knowledge and understanding about diabetes risk and preventive measures particularly in the low-income community; supporting lifestyle modification (e.g., upskilling, goal setting, action planning) in both communities; identifying people at high risk of diabetes through screening in both communities; and creating social and physical environments for lifestyle modification (e.g., positive social influences on healthy living, access to healthy foods and physical activity infrastructure) particularly in the low-income community. Residents from both communities agreed that the strategies were broadly feasible for diabetes prevention but proposed the addition of door-to-door campaigns and community theatre for health education. However, residents from the low-income community were concerned about the lack of government prioritisation for implementing population-level interventions, e.g., improving access to healthy foods and physical activity facilities/infrastructure.

**Conclusion:**

Diabetes prevention initiatives in Kenya should involve multicomponent interventions for lifestyle modification including increasing education and upskilling at individual level; promoting social and physical environments that support healthy living at population level; and are particularly needed in low-income communities.

## Introduction

Diabetes is a major global health problem [1]: in 2021, it caused 6.7 million deaths worldwide and was associated with a health expenditure of over US $960 billion [2]. The prevalence of diabetes is rising and is forecast to increase by 46% by 2045 [2]. Type 2 diabetes, which accounts for 90–95% of diabetes cases, isto a large extent, preventable through lifestyle changes such as weight control, healthy eating, and increased physical activity [1].

The rising burden of diabetes will increasingly fall on Sub-Saharan Africa where its prevalence is projected to rise 2.5-fold between 2021 and 2045, and the associated health expenditure from US $12.6 to US $ 46.7 billion [2]. Kenya has recognised diabetes as a major public health problem since 2015 [3]. A national population survey showed the prevalence of type 2 diabetes was almost two times higher in urban (3.4%) compared to rural (1.9%) areas [4]. Other surveys reported even higher prevalence in low-income urban communities in the Kenyan capital, Nairobi (4.1–5.3%) [5, 6]. Diabetes-related deaths in Nairobi were estimated to have increased by 65% between 2009–2019, and diabetes was ranked among the top 10 causes of death and disability in Kenya in 2019 [7].

The high diabetes burden in Nairobi could be due to two reasons. First, delayed diabetes diagnosis may contribute to increased diabetes complications and mortality. A national survey found that the prevalence of undiagnosed diabetes was 51% in urban settings [4], while a cross-sectional study of 50 patients attending a primary health facility in Nairobi found that 52% had undiagnosed diabetes [8]. Second, there could be a high diabetes incidence due to increased exposure to diabetes risk factors including excess weight, unhealthy diet, and physical inactivity [9]. In 2015, the prevalence of excess weight (overweight or obesity) was 38% in Nairobi, which is substantially higher than the national average (28%) [10]. In addition, the majority (~60%) of Nairobi residents do not eat the recommended daily servings of fruit and vegetables [11], with the unaffordability of healthy foods being reported as a major barrier to healthy eating [12]. Furthermore, although self-reported physical activity levels are generally high in Kenya, with only ~8% of Kenyans not meeting the recommended levels, more urban residents tend to be physically inactive than rural residents (~11% versus 6%) [13]. There is therefore an urgent need to develop preventive interventions targeting modifiable lifestyle factors to reduce the incidence and consequently the rising burden of diabetes in urban settings.

Careful development of interventions informed by local contextual factors is necessary to increase their likelihood of effectiveness and avoid wasting public resources [14, 15]. However, a recent analysis of Kenyan diabetes prevention and control policies found that although strategies were well aligned to international recommendations, they did not reflect the local context and required more locally-generated evidence to inform tailored prevention measures [16]. Additionally, a recent qualitative study on priority-setting for noncommunicable disease (NCD) control in Kenya has called for context-specific evidence on effective local interventions [17]. To contribute to context-specific evidence, there is need to apply a people-centred approach (one of the guiding principles of the Kenya NCD Strategic Plan), which acknowledges the need for community involvement and participation in selecting, implementing, and monitoring interventions [18]. Therefore, this study aimed to explore diabetes risk and co-create a diabetes prevention theory of change in two socioeconomically distinct communities to inform future local diabetes prevention interventions.

## Methods

The current study was guided by the steps two and three of the 6SQuID (six essential Steps for Quality Intervention Development) model for quality intervention development [15] and was conducted in two phases: Phase 1 (6SQuiD step two) identifying modifiable determinants (in-depth interview study); and Phase 2 (6SQuiD step three) identifying the mechanisms of change (theory of change development).

### Phase 1

Phase 1 aimed to 1) explore the contexts, perceptions, barriers, facilitators, and opportunities for lifestyle changes to reduce diabetes risk in two socio-economically distinct communities in Nairobi, Kenya; 2) conceptualise the impact and long-term outcomes that would guide the theory of change development in Phase 2.

#### Study design, population, and setting

A qualitative case-study design was used to conduct an in-depth investigation into local perceptions of diabetes risk [19, 20]. The study population was people without diabetes aged 35–60 years, as diabetes risk is highest in this age group in Kenya [4]. Participants were recruited from Mukuru (a low-income community) and Buruburu (a middle-income community). Mukuru is one of the largest informal settlements in Nairobi and is located near the city’s main industrial area which provides employment (mainly as casual labourers) to many local people [21, 22]. Buruburu is a nearby residential area established in post-colonial Kenya to promote house ownership [23].

#### Sampling and recruitment

Purposive sampling was used to recruit men and women aged 32–58 years in each community. To strike a balance between a pragmatic approach [24] and achieving data saturation, a target of 32 participants (16 (8 women) from each community) was set. At the end of the study period, 29 participants were recruited: 15 (7 women) in Mukuru and 14 (6 women) in Buruburu. Most participants (n = 21) were identified through community gatekeepers, and the rest (n = 8) by asking participants to refer friends interested in taking part in the study.

#### Data collection and analysis

Data were collected between March-July 2020 using semi-structured face-to-face (n = 5) and telephone (n = 24) interviews in either English (n = 8) or Swahili (n = 21). Interviews were audio-recorded, transcribed, and translated into English if conducted in Swahili. Interviews lasted about 40 minutes (mean: 39, range 22–55 minutes in Mukuru; and 44, range 26–72 minutes in Buruburu). A topic guide (see S1 File) exploring participants’ perceptions of diabetes, weight, diet, and physical activity was used to guide discussions. Transcribed and anonymised interviews were imported to NVivo 12 and analysed thematically [25]. Two authors (AMM and CMG) independently read five transcripts and met to agree on the coding frame, which AMM then applied to all transcripts.

Themes relating to the barriers and facilitators of lifestyle modification were mapped on the COM-B model of the Behaviour Change Wheel [26]. In COM-B, Capability is defined as an individual’s psychological (including the necessary knowledge and skills) and physical capacity to engage in behaviour. Opportunity is the social and physical environment that prompts or enables behaviour. Motivation describes the brain processes that stimulate and guide behaviour, and includes reflective (goals, analytical decision-making) and automatic (habits, emotional stimulants) motivation [26].

### Phase 2

Phase 2 aimed to co-create with community residents a theory of change for diabetes prevention in Nairobi, Kenya.

#### Structure

The original study design involved a series of interactive co-creation face-to-face workshops with community residents. However, the COVID-19 restrictions in place in Kenya in early 2021 prevented face-to-face meetings. Instead, the research team used the Phase 1 findings to develop a diabetes prevention theory of change using an modified four-stage approach [27–29]: 1) conceptualisation of preconditions; 2) outlining interventions, assumptions, rationale, resources and stakeholders; 3) visual presentation and narrative write up; and 4) quality review and input. One author (AMM) carried out steps 1 to 3 and two authors (CMG and EM) contributed to step 4, which also involved consultations with a subset of Phase 1 participants, who provided input on the feasibility and suggested additions needed on the developed theory of change.

#### Sample

Twenty residents from the two Nairobi communities who had participated in the Phase 1 interviews were purposively selected and invited to review the theory of change. Eight participants (3 female) from Mukuru and five participants (1 female) from Buruburu (total n = 13) were able to participate by the end of the study period.

#### Quality review and input on the theory of change data collection

Data were collected between January-March 2021 using telephone interviews. The interviews lasted about 20 minutes (mean: 23, range 17–32 minutes), and the majority (10/13) were conducted in Swahili.

Presenting participants with a complex visual diagram of the theory of change outwith the context of a face-to-face workshop might have limited their ability to provide meaningful input. Therefore, four narrative vignettes (short hypothetical stories) [30] summarising the theory of change for diabetes prevention were developed–see in S1 Fig. Each vignette was developed in English and translated into Swahili, and participants were asked if they preferred the English or Swahili versions. Vignettes were shared with participants as hard copies or electronically via email or WhatsApp messenger dependent on their preferences. After providing participants with time to read through all the four vignettes, a short telephone interview was conducted to discuss at least two vignettes, with participants commenting on their plausibility, feasibility, and any additions or modifications that should be made. To ensure all vignettes were discussed, the first was chosen by the participant and the other by AMM. Interviews were audio recorded, detailed notes taken and summarised, and some parts were transcribed to provide supporting extracts.

Supporting extracts from findings are presented with participants’ gender, age-group, profession, and community (e.g., *M*, *40–49*, *Businessman*, *Buruburu* is a male businessman aged 40–49 years from Buruburu).

### Ethical considerations

Ethical approvals were obtained from the Great Lakes University of Kisumu Research Ethics Committee, ID GREC 023/19, Kenyatta National Hospital–the University of Nairobi Ethics and Research Committee, P383/05/2019, and the University of Glasgow College of Medical, Veterinary and Life Sciences Ethics Committee, project 200180144. Conduct of the research was also approved by the National Commission for Science, Technology, and Innovation in Kenya. Informed consent (written for face-to-face interviews or verbal for telephone interview) was sought from all participants before data collection.

## Results

### Phase 1

As shown in Table 1, the Phase 1 interviewees were middle-aged (~44 years in Mukuru and ~41 years in Buruburu). The majority in Mukuru (9/15) were only educated to primary level, while most in Buruburu (10/14) had tertiary level education. Being a businessperson was the most reported profession among participants in both communities; other professions included shopkeeper and banker (in both communities), Information Technology and real estate consultants (in Buruburu), and two people in Mukuru were unemployed.

**Table 1:** In-depth interview participant characteristics in each community.

	Mukuru (n = 15)	Buruburu (n = 14)
**Age (years)–mean ± SD**	44.4 ± 7.0	40.9 ± 6.5
**Gender–n (%)**		
Women	7 (47)	6 (43)
Men	8 (53)	8 (57)
**Education level–n (%)**		
Primary	9 (60)	0 (0)
Secondary	3 (20)	4 (29)
Tertiary	3 (20)	10 (71)
**Profession–n (%)**		
Businessperson	4 (27)	5 (36)
Consultant	0 (0)	2 (14)
Shopkeeper	2 (13)	1 (7)
Carpenter	1 (7)	0 (0)
Media producer	0 (0)	1 (7)
Taxi driver	0 (0)	1 (7)
Unemployed	2 (14)	0 (0)
Pharmacist	1 (7)	0 (0)
Hairdresser	1 (7)	0 (0)
Banker	1 (7)	1 (7)
Tea lady	1 (7)	0 (0)
Cleaner	1 (7)	0 (0)
Casual labourer	1 (7)	0 (0)
Social worker	0 (0)	1 (7)
Mechanic	0 (0)	1 (7)
Human resource officer	0 (0)	1 (7)

SD–standard deviation

### Perceptions of diabetes and diabetes risk factors

There were contrasting perceptions of the most important health problems in the two communities. In Mukuru, most residents mentioned communicable diseases (e.g., HIV/AIDS, malaria, tuberculosis) and health-related issues (e.g., alcohol abuse, poor water and sanitation, air pollution, poverty, inaccessibility of healthcare services). However, some Mukuru participants also noted that NCDs, including diabetes, were increasing. In contrast, Buruburu residents reported that NCDs (diabetes, hypertension, cancer, arthritis, asthma, gout) were the leading health problems in both young and old generations, while communicable diseases were less of a concern:

*Because most of our parents who are the owners of the houses in Buruburu are mainly people who bought the houses back in the ‘80s, so it is only like diabetes is really, really affecting them right now. If you compare with other areas, most old people who are in their 60s right now live in Buruburu, yeah? Nevertheless, there is a group of young people who are generally experiencing the same diseases like their parents in terms of high blood pressure, diabetes, some, cancers here and there. But generally, in Buruburu there is no such concern like cleanliness, or bilharzias, cholera, there is clean water in Buruburu. [M, 30–39, Media Producer, Buruburu]*

Regarding diabetes risk factors, several participants from both communities identified excess weight as increasing in their communities (although some Mukuru residents thought that poverty meant weight gain was less of an issue locally). They suggested this was particularly the case for women and the middle-aged, although some had also noticed increased weight among children. Some Buruburu participants felt that excess weight was contributing to NCDs, such as diabetes.

*Guys of my age one of the key problems that they suffer from is overweight which then either triggers diabetes or triggers hypertension. [M, 50–59, Consultant, Buruburu]*

With regard to diet, most Mukuru residents reported that they did not eat fruit and vegetables daily. Interviewees from both communities felt that sugar intake was high locally, and that people were eating the unhealthier westernised diets which were becoming readily available:

*You know our days [when I was younger] we used to eat healthy food. These days junk food is all over. For example, chips, bhajias [Swahili potato fritters], you know? Biscuits, crisp, they are all over [F, 50–59, Tea-lady, Buruburu]*

Finally, although residents from both communities felt that physical activity, especially leisure-related physical activity, was perceived more positively than a decade ago, they recognised that activity levels were lower than they had been in the past:

*I think that long ago people were more active, currently, people are not exercising a lot, they are very sedentary, they are more indoors, more than long ago. [M, 40–49, Carpenter, Mukuru]*

### Barriers and facilitators of lifestyle modification

The analysis identified four main themes corresponding to the long-term outcomes (LTO) required for the prevention of diabetes: LTO 1—understanding personal diabetes risk; LTO 2—engaging in weight control; LTO 3—eating a healthy diet; and LTO 4—engaging in physical activity. Table 2 shows a summary of the barriers and facilitators related to the four LTOs mapped onto the COM-B model constructs: capability (psychological and physical), opportunity (social and physical), and motivation (reflective and automatic).

**Table 2:** Long term outcomes and the associated barriers and facilitators of achieving them mapped onto COM-B constructs.

Long-term outcomes (LTO)	Barriers (X)/Facilitators (√) [community where this was most common] *	Effect on COM-B constructs
LTO 1. Understanding personal diabetes risk	1.1 Limited knowledge of diabetes and diabetes risk (X) [M]	**↓**Psychological Capability
	1.2 Limited practical knowledge about how to prevent diabetes (X) [M]	**↓**Physical Capability
	1.3 Low disclosure of diabetes by people with the condition (X) [M]	**↓**Social opportunity
	1.4 Limited availability and affordability of diabetes screening services (X) [M]	**↓**Physical opportunity
LTO 2. Engaging in weight control	2.1 Limited knowledge about what constitutes a healthy weight (X) [both]	**↓**Psychological Capability
	2.2 Lack of weight control skills (X) [M]	**↓**Physical Capability
	2.3 Negative societal norms around weight control (X) [both]	**↓**Social Opportunity
	2.4 Limited understanding of the health consequences of excess weight (X) [M]	**↓**Reflective Motivation
	2.5 Understanding the benefits of having a lean body mass (√) [B]	**↑**Reflective Motivation
	2.6 Fatalist views about weight gain [M]	**↓**Reflective Motivation
	2.7Weight loss plans (√) [B]	**↑**Reflective and Automatic Motivation
LTO 3. Eating a healthy diet	3.1 Limited knowledge about (un)healthy eating (X) [both]	**↓**Psychological Capability
	3.2 Social influences (including during childhood) promoting eating of unhealthy diets (X, √) [both]	**↕**Social Opportunity
	3.3 Limited availability and affordability of healthy foods (X) [M]	↓Physical Opportunity
	3.4 Perceived safety concerns about some healthy foods (e.g., vegetables) (X) [both]	**↓**Reflective and Automatic Motivation
	3.5 Increased understanding of the benefit of healthy eating (√) [both]	**↑**Reflective Motivation
	3.6 Need to make food tastier (X) [both]	↓Automatic Motivation
	3.7 Meal plans (√) [B]	**↑**Reflective and Automatic Motivation
LTO 4. Engaging in physical activity	4.1 Limited knowledge of physical activity recommendations (X) [both]	**↓**Psychological Capability
	4.2 Perceived time constraints (X) [both]	**↑**Physical Opportunity
	4.3 Societal perceptions that undermine physical activity (X) [M]	**↓**Social Opportunity
	4.4 Positive societal perceptions towards physical activity (√) [B]	**↑**Social Opportunity
	4.5 Exercising together (√) [both]	**↑**Social Opportunity
	4.6 Limited access (availability or affordability) to physical activity facilities or infrastructure (X) [M]	**↓**Physical Opportunity
	4.7 Understanding the benefits of physical activity (√) [both]	**↑**Reflective Motivation
	4.8 Fatalist views on impact of physical activity [B]	↓Reflective Motivation
	4.9 Physical activity in childhood leading to habit formation in adulthood [B]	**↑**Automatic Motivation

*M = Mukuru; B = Buruburu

↑ = Increase; ↓ = Decrease

### Capability

A cross-cutting barrier across the long-term outcomes was limited knowledge reducing psychological capability for lifestyle modification. For example, most participants from both communities were unaware whether their current weight was healthy or not. Furthermore, none of the participants were aware of how much physical activity they should be doing each week nor of the daily recommended servings of fruit and vegetables:

*No, I don’t know [recommended servings of fruits and vegetables]. I only buy kales for 30 shillings, or even those traditional vegetables for 30 shillings, just that. Mmm but I do not know what portion I am supposed to eat. [F, 30–39, Unemployed, Mukuru].*

Apart from knowledge, limited skills were perceived to be another barrier to lifestyle modification in Mukuru. Limited skills for weight loss and diabetes preventive measures were reported by many participants, including those with personal experiences of diabetes:

*I do not know what to say about prevention because even my father died because of diabetes, and I do not know what a person can do for sure to prevent diabetes. Not unless I get to know what a person can do about diabetes, I can understand about it then, eeh but I am not sure of what can be used to prevent diabetes. [F, 40–49, Hairdresser, Mukuru]*

### Opportunity

Social opportunity was mentioned as an important barrier or facilitator for all four long-term outcomes. A perceived barrier of understanding personal diabetes risk was people with diabetes failing to disclose their condition, which meant a missed opportunity for others to become aware of diabetes and reflect on their possible risk exposures. There were interesting differences between the two communities. While some interviewees in Buruburu felt that people could disclose their diabetic status quite easily, the majority of participants from Mukuru felt that this was not the case due to fear of stigma and social isolation:

*Many people think of it as a disease that is not good, so, they feel as if they will be stigmatized and segregated. That is why you see people fearing this, they take it like being HIV positive and so they will fear to openly say this, and it makes a person stay with their problems. [M, 40–49, Carpenter, Mukuru]*

Other important perceived influences were social norms towards (un)healthy lifestyles. Negative influences included: associating weight loss with illness (such as HIV) or financial hardship; use of unhealthy foods (processed or high in sugar and fats) as treats or rewards; associating western (unhealthier) diets with high socioeconomic status, and traditional (healthier) diets with low socioeconomic status; associating exercise with young people; and associating walking with financial hardship. However, participants reported that such ‘traditional’ norms were gradually being replaced by more positive perceptions. These included lean bodies being viewed as attractive; and walking being valued as a form of physical activity and not seen as showing lack of wealth (particularly in Buruburu):

*I told you that currently, people think about it as exercise, even walking as a form of exercise. They will not think that you do not have money for using the car, or for a motorbike, or for paying Uber. It is a form of exercise. [F, 30–39, Businesswoman, Buruburu]*

Apart from social norms, friends and community members were mentioned as positive influences on lifestyle changes through encouraging weight loss, healthy eating, and physical activity, mainly in Buruburu:

*I can say it is the discussions, eeh, discussion, it becomes live [frank]. There is not much beating about the bush, people are honest. “Hey mum, [salutation to an older woman], how are you thinking about that weight?”, some people will not take it kindly, but at least you have said it. You will not sugar-coat it; you will tell them, “My friend, that weight, man, what is happening?” A person will accept this and say, “ha, by the way, I am heavy, I need to do something”. [M, 40–49, Businessman, Buruburu].*

Furthermore, exercising together (with friends and/or in a team) was reported as a positive influence on physical activity in both communities. An example of this was a women’s group in Mukuru, which, besides being a money-saving group, was a football team:

*We meet every Saturday, at 2 pm; we meet in the field at the community field near the police post. That is where we play the women’s games and we even go to competitions. Mmm, we are many women; we are more than 100. On Saturday we also do save money; Kshs. 100 each, eeh. [F, 40–49, Businesswoman, Mukuru]*

Physical opportunity (time and resources) was reported as an important influence on understanding personal diabetes risk and lifestyle modification. Some Mukuru residents noted that most local people had not been tested for diabetes due to unavailability or unaffordability of diabetes testing at local health facilities. However, even when testing could be accessed, there was fear of despair associated with a positive diagnosis due to the perceived cost of managing diabetes:

*In my opinion, not many people have gone for testing because of fear. You can find a person going to test and then you find that you have it [diabetes]. You see now that is a problem that has come in and you will start thinking, “I now have this disease. What will I use to treat it with?, I do not have money” so many have failed to go for testing because of that fear. [F, 30–39, Unemployed, Mukuru]*

In both communities, (un)availability and (un)affordability of healthy or unhealthy foods influenced healthy eating and consequently weight control. Unavailability of seasonal fruit and vegetables was a barrier to healthy eating in both communities, whereas perceptions of unaffordability differed: in Buruburu, participants complained about the cost of meat; while in Mukuru, fruit and vegetables, as well as meat, were viewed as unaffordable. Residents in both communities reported the widespread availability and affordability of fast foods as a barrier to healthy eating. These included local street foods in Mukuru and from restaurants and fast-food franchises in Buruburu:

*We will go back to the same point; we have KFC [Kentucky Fried Chicken] in Buruburu, and people will go there after every two days to buy KFC. In KFC there is a package for 210 [~£2], there is a package for 340 [~£3], so, they can afford. So, you see, that contributes to that [intake] because of the affordability. [M, 40–49, Businessman, Buruburu]*

Limited availability (mainly in Mukuru) and affordability (in both communities) of physical activity facilities (e.g., gymnasiums, sports fields) was considered to be a barrier to people engaging in physical activity and using it as a way of controlling their weight. Some participants blamed corruption (“land grabbing”: privatisation of public land meant for community use) for the lack of local sports fields:

*There is an issue in Buruburu on concerns of public land being grabbed. Yeah? Playgrounds being grabbed [M, 30–39, Media producer, Buruburu]*

Affordable motorized transport (Uber taxis in Buruburu and motorbike taxis *[boda boda]* in Mukuru) was perceived to hinder physical activity (active travel) in both communities. Additionally, infrastructure was reported to be poor in Mukuru: here lack of sidewalks; open drainage channels and mud during rainy seasons; and high rates of crime made walking difficult and unsafe:

*Walking on foot is also risky, especially in Kenya where there are no footpaths for walking or jogging. The road is small, the motorbikes are there, vehicles are overlapping, it is not secure. This is not like in other countries where you see that there is a footpath set aside, and even if you are walking or jogging, you will not be on the same road as the motorbikes or bicycles. That is a challenge because accidents happen. Many people are afraid at the moment [of walking]. Some fear going to the road they will be attacked; they will be mugged. [M, 40–49, Carpenter, Mukuru]*

Finally, time constraints due to competing interests, such as work and family responsibilities, and going to church, were identified as common barriers to physical activity in both communities.

#### Motivation

Understanding the benefits of leading a healthy lifestyle and the consequences of having an unhealthy lifestyle were mentioned as reflective motivators for lifestyle change in both communities. One woman from Mukuru recounted how knowing the harmful effects of unhealthy foods and seeing the consequences of unhealthy eating (such as in people with diabetes) encouraged her to improve her diet:

*Long ago I was drinking a lot of that [added sugar in beverages]. I would stir a lot of it. Eeh but I stopped doing that. Now you know when you get some little knowledge that this thing will be harmful, you stop, and when you see how someone is struggling with diabetes, you just keep off because you do not want to get into that trap. [F, 30–39, Businesswoman, Mukuru]*

However, residents from both communities felt that a need to make food tastier by adding sugar, fats, and oils led to many people eating unhealthily. Some participants also felt that an excessive intake of sugary foods led to decreased sweet sensitivity, which made it hard for people to reduce their sugar intake, as recounted by one woman:

*They [people who like sugar] normally say that their ‘mouth has gone’ that is why they like a lot of sugar. [F, 40–49, Businesswoman, Mukuru]*

Some participants from both communities described how fatalist views, such as excess weight being God-given, prevented people being motivated to lead a healthy lifestyle:

*So, that one [having excess weight] now becomes a challenge because you do not know what happens; it is God who has planned. I sometimes think it is God who has a hand in it. [F, 30–39, Businesswoman, Mukuru]*

People’s diet and physical activity were perceived by residents in both communities to reflect habits formed during childhood (automatic motivation). For example, one man from Buruburu described how his current attitude towards food was influenced by the fact that healthy eating was not a priority in his family when he was a child:

*We were never brought up being cautious about, making sure that any time you take aah a meal, it has to be a balanced meal. We were always brought up thinking that whatever is on the table is whatever you take, and you are good to go. [M, 40–49, Social worker, Buruburu]*

Concerns about food safety, such as contamination during growth, storage, or preparation for sale, including the overuse of farm chemicals (fertilizers, pesticides) and artificial ripening of fruits, were also reported as preventing people from eating a healthy diet. One woman from Buruburu described how she was put off eating vegetables because of concerns about the conditions in which those sold locally were grown:

*I have doubts about the vegetables because in most cases we are not sure where they come from. Yeah, there is this perception that especially kale and spinach have been planted, okay, in most cases we believe they come from sewages. [F, 30–39, Human Resource Officer, Buruburu]*

Finally, a few participants from Buruburu suggested that providing people with weight loss and meal plans would motivate and support them to lead healthier lifestyles.

### Phase 2 results

#### Conceptualisation of preconditions

Based on the identified barriers and facilitators (in Table 2), 10 preconditions (i.e., requirements or strategies) needed to achieve the four LTOs were identified–see Table 3. Precondition 1 − increased knowledge and understanding about diabetes and diabetes risk, and Precondition 2 − improved societal perceptions towards diabetes and lifestyle modification were crosscutting in all four LTOs. To complement Precondition 1, specifically in relation to LTO 1 (increasing understanding of personal diabetes risk), Precondition 3 –involvement of people with diabetes, as local “diabetes prevention champions”, was conceptualised. For LTOs 2 (engaging in weight control), 3 (healthy eating), and 4 (physical activity), improved skills for lifestyle change (Precondition 4), and increased support for healthy living through goal setting e.g., through weight loss plans, meal plans, physical activity goals (Precondition 5) were conceptualised. Precondition 6 (increasing screening in communities) included diabetes screening for LTO 1 and central obesity screening for LTO 2. Preconditions 7 (increased availability and affordability of healthy foods) and 8 (improved food safety) related to LTO 3 (eating healthy diets). Finally, Preconditions 9 (increased support for group-based physical activity) and 10 (increased availability, affordability, and usability of physical activity infrastructure and facilities) contributed to LTO 4 (engaging in physical activity).

**Table 3:** Preconditions, relevant LTOs, functions, BCTs, and rationale to achieve the four LTOs.

Preconditions needed [community where most needed] *	Relevant LTO	BCW intervention/policy FUNCTIONS	BCTs incorporated in the functions	COM-B rationale for change
1) Increased knowledge and understanding about diabetes and diabetes risk (M) 2) Improved societal perceptions towards diabetes prevention [M]	LTO 1, 2, 3, 4	EDUCATE/COMMUNICATE about diabetes and diabetes risk (e.g., health consequences of diabetes, excess weight, unhealthy eating, physical inactivity, preventive measures)	• Information on health consequences	Increased psychological capability, reflective motivation, and social opportunity
		PERSUADE about consequences of diabetes and unhealthy lifestyles and benefits of preventive measures (e.g., weight control, healthy eating, physical activity)	• Pros and cons • Comparative imagining of future outcomes • Salience of consequences	
3) Involvement of people with diabetes in increasing local knowledge [M]	LTO 1	ENABLE and TRAIN people with diabetes to be “diabetes prevention champions” in their community	• Information the health and social consequences • The salience of consequences	Increased reflective motivation
4) Increased diabetes (M) and central obesity screening in communities [both]	LTO 1, 2	PROVIDE SERVICES − diabetes and central obesity screening	• Biofeedback	Increased reflective motivation
5) Improved skills for lifestyle modification [both]	LTO 2, 3, 4	EDUCATE about weight control, healthy diet composition, muscle strengthening. TRAIN in weight control methods, healthy diet preparation, muscle strengthening	• Instruction on how to perform the behaviour. • Demonstration of behaviour	Increased psychological and physical capability
6) Increased support for lifestyle changes (i.e., weight control, healthy eating, physical activity) [both]	LTO 2, 3, 4	TRAIN and ENABLE development of weight loss plans, meal plans, or physical activity goals	• Action planning • Goal setting • Self-monitoring of behaviour	Increased reflective and automatic motivation
7) Increased availability and affordability of healthy foods [M]	LTO 3	ENVIRONMENTAL PLANNING and FISCAL policies to ensure availability and affordability of healthy foods	• Restructuring the physical environment • Adding objects to the environment	Increased physical opportunity
8) Improved food safety [both]	LTO 3	REGULATE to ensure food safety	• Remove aversive stimulus	Increased reflective and automatic motivation
9) Increased support for group-based physical activity [both]	LTO 4	ENABLE people to exercise in groups	• Social support	Increased social opportunity
10) Increased availability and affordability of physical activity facilities [both] and infrastructure [M]	LTO 4	ENVIRONMENTAL PLANNING to provide facilities and infrastructure	• Adding objects to the environment	Increased physical opportunity

*M = Mukuru

BCT–Behaviour Change Technique; BCW–Behaviour Change Wheel; COM-B–Capability, Opportunity, and Motivation as sources of Behaviour model

LTO–Long-term outcome: LTO 1 –increased understanding of personal diabetes risk; LTO 2 –increased engagement in weight control; LTO 3 –increased eating of healthy diets; and LTO 4 –increased engagement in physical activity.

To achieve these preconditions four intervention and five policy functions from the Behaviour Change Wheel were identified (see Table 3). The intervention functions were: education (for Precondition 1, 2, 4); persuasion (Precondition 1, 2); training (Precondition 3, 5, 6); and enablement (Precondition 6, 9). The policy functions included: communication (Precondition 1, 2); provision of services (Precondition 4); environmental planning (Precondition 7, 10); regulation (Precondition 8); and fiscal measures (Precondition 7).

The Behaviour Change Technique (BCT) Taxonomy (v1) [31] was used to specify the intervention and policy functions (i.e., the active ingredients). As shown in Table 3, 15 BCTs were identified: information on health consequences (for education intervention function); comparative imagining of future outcomes (persuasion); demonstration of behaviour (training); goal setting (enablement); social support (enablement); biofeedback (for providing services policy function); restructuring the physical environment (environmental planning); adding objects to the environment (environmental planning and fiscal measures).

#### Outlining interventions, assumptions, rationale, resources, and stakeholders

Intervention examples that would lead to achievement of preconditions were identified based on evidence of interventions that had worked in other settings or for other health conditions. In total, as shown in Table 4, 12 intervention examples were outlined. The intervention examples were diverse, targeted various preconditions and included: education through television drama; diabetes risk and central obesity screening by community health volunteers; cookery demonstrations; kitchen gardens; group exercising; and community-run recreation facilities among others.

**Table 4:** Intervention examples and associated preconditions along with supporting evidence for their choice.

Intervention example [community where most needed] *	Rationale	Associated Preconditions (see footnote)
1. A television drama programme to increase knowledge and understanding about personal diabetes risk, weight, diet, and physical activity [M]	• Knowledge and understanding increases psychological capability and reflective motivation based on COM-B model [26] • In HIV/AIDS research, more knowledge associated with lower levels of stigma in Kenya [32]	Preconditions 1 and 2
2. Facilitating people with diabetes to become ‘diabetes prevention champions’ who would increase local knowledge about diabetes in communities [M].	• Increasing local knowledge on diabetes risk: disclosure by people with HIV/AIDS was associated with an eagerness to know about prevention among uninfected [33]	Precondition 3
3. Trained community health volunteers conduct a door-to-door campaign to identify people at high risk of diabetes [both]	• Intervening in people at high risk more cost effective than whole population, therefore, targeted screening in people at high risk (identified using risk scores and biochemical tests^1^) more appropriate [34, 35].	Precondition 4
4. Trained community health volunteers conduct door-to-door central obesity screening [both].	• Central obesity is a better predictor of diabetes risk than general obesity in this population^2^ [36, 37]	
5. Trained community health volunteers educate, and train people found to be centrally obese in weight loss methods (e.g., diet and exercise) and facilitate them to develop personal weight loss plans [both].	• Skills increase the physical capability based on COM-B model [26] • Action planning and goal setting contributes to reflective motivation and, with time, automatic motivation (through habit formation) for healthy living [26] • Inclusion of muscle-strengthening exercises based on evidence that diabetes is associated with low muscle strength in this population [36].	Preconditions 5 and 6
6. Cookery demonstration to develop healthy meal plans using local foods [both].		
7. Trained community health volunteers train people in home-based muscle strengthening and other exercises and facilitate them to set achievable physical activity goals [both].		
8. Support people to grow their food, such as through kitchen gardens [both]	• Based on COM-B model [26], availability and affordability of healthy feeds increases the physical opportunity of healthy eating^3^	Precondition 7
9. Community markets that benefit from healthy food subsidies to improve affordability throughout the year [M]		
10. Local public health departments to regulate the safety of all foods in markets [both]	• Alleviates concerns on food safety contributing to automatic motivation [26] of healthy eating	Precondition 8
11. Provision and facilitation of group-based exercises in existing community groups (e.g., women savings groups) or workplaces [both]	• Increases the social opportunity for engaging in physical activity drawing from the COM-B model [26]	Precondition 9
12. Community-based organisation run low-cost recreation facilities (sports fields and gymnasiums) [both]; and sidewalks [M]	• The intervention contributes to the physical opportunity [26] of engaging in physical activity	Precondition 10

*M = Mukuru

Superscripts

Assumption is that the risk score questionnaire and biochemical tests used has high validity for the Kenyan population.

Assumption that the international central obesity cut-offs are valid for the Kenyan population.

Assumption that healthy foods are available and affordable throughout the year.

**Preconditions:** 1) Increased knowledge and understanding about diabetes and diabetes risk; 2) Improved societal perceptions towards diabetes and diabetes risk; 3) Involvement of people with diabetes in increasing local knowledge; 4) Increased diabetes and central obesity screening in communities;5) Improved skills for lifestyle modification; 6) Increased support for lifestyle changes (i.e., weight control, healthy eating, physical activity); 7) Increased availability and affordability of healthy foods; 8) Improved food safety; 9) Increased support for group-based physical activity; 10) Increased availability and affordability of physical activity facilities and infrastructure

The assumptions, contextual conditions needed for the change pathway not to be broken, were: first, the tools used in diabetes risk screening in Intervention 3 have high validity for the local population. Second, the central obesity cut-offs used in Intervention 4 are valid for the Kenyan population. Third, the foods included in the meal plans (Intervention 6) will be available and affordable throughout the year. Additionally, Interventions 6, 8, 9, and 10, could contribute to the formation of healthy eating habits in childhood (and thus in future adulthood) through improving family meals assuming that children are not exposed to unhealthy diets, which may contribute to habit reversal, in other settings (e.g., schools). Rationale for the choice of intervention examples was based on relevant scientific evidence as outlined in Table 4. Effectiveness of similar intervention examples used in other settings or diseases is provided in Table 1 in S1 File.

Finally, to realise the intervention examples, various resources and stakeholders are needed. Funding was the main resource requirement but spreading knowledge (increasing awareness) throughout the community would also be important. Awareness-raising resources might include settings (churches, schools, health facilities), mechanisms (mass/social media), or people (health workers, local support groups (e.g., diabetes support groups)). Stakeholders were identified as governments (county and national), development partners (such as non-governmental organisations), local communities (including potential end-users and local leaders), and community-based organisations.

#### Narrative write-up and visual presentation

This step was done simultaneously with the preceding two steps which have described all components of the theory of change. Four figures were used to visually present separate theories of change for each of the four long-term outcomes, see in S1 Fig. Each theory of change was then developed into a vignette (see in S1 Fig), which was then presented to community members for review and input.

#### Quality review and input

The mean age of participants in this review phase was 39.8 ± 5.8 years, almost half (6/13, 46%) had tertiary-level education, and most (8/13, 62%) were self-employed. Table 5 shows the number of participants discussing each vignette (some participants chose to discuss more than two vignettes).

**Table 5:** Number of participants who reviewed each of the four vignettes.

Vignette	Participant choice	Author allocation	Third choice	Total
1: Understanding personal diabetes risk	6	1	1	8
2: Engaging in weight control	2	4	1	7
3: Eating healthy diets	2	5	1	8
4: Engaging in physical activity	3	3	1	7

Generally, residents from both communities agreed that the proposed interventions would contribute to diabetes prevention and felt the most impactful ones included: increased knowledge (about diabetes, weight, diet); door-to-door campaigns to identify people at high risk of diabetes and to conduct obesity screening; support in the form of improved skills (in healthy diet preparation and muscle strengthening) and development of meal plans; increased access to healthy foods and physical activity facilities; and exercising as a group–see Box 1.

**Box 1. Most impactful interventions/preconditions for diabetes prevention reported by participants in order of most to least reported for each of the four vignettes**

**Vignette 1: Increasing understanding of personal diabetes risk**

- Door-to-door campaigns to identify people at high risk
- Increased knowledge about diabetes

**Vignette 2: Increase in engagement in weight control**

- Door-to-door central obesity screening
- Increased knowledge about a healthy weight

**Vignette 3: Increase in eating of healthy diets**

- Increased skills in healthy diet preparation
- Support in developing meal plans
- Increased affordability of healthy foods
- Increased knowledge about a healthy diet
- Increased urban farming through kitchen gardens

**Vignette 4: Increase in physical activity levels**

- Facilitating exercising as a group
- Demonstration of muscle-strengthening exercises
- Access to physical activity facilities

Interviewees were prompted to suggest any challenges associated with the interventions proposed, modifications, or other interventions that should be considered. A key concern for some participants from Mukuru was that population-level interventions (e.g., access to healthy foods and physical activity facilities/infrastructure) were unlikely to be realised due to government corruption and lack of prioritisation for their implementation:

*They [physical activity facilities and infrastructure] can help in a big way, but you know this government does not care, so we cannot really depend on it. What we can depend on is walking on the road as normal, if you have your bicycle, you cycle it in the neighbourhood because if we wait for the government to construct [sports] fields and good roads to use, it will not happen, and people will die as we wait. So, we work with what is available [M, 30–39, Shopkeeper, Mukuru]*

While some participants felt that kitchen gardens would improve food access and ensure food safety, there were concerns by some residents from both communities that they might not be practical in some contexts (e.g., in rental houses). Additionally, participants from both communities questioned the effectiveness of a television drama as some (mainly low-income) households did not own a television). It was suggested that door-to-door education by community health volunteers could reach more people and promote better engagement through one-to-one interaction. Alternatively, one businessman suggested the drama could be performed at a community theatre to ensure that no-one missed out:

*You see, because not everyone has access to the TV so what will people do there? So, that is where you can have NGOs can go in and can even create a stage in the community, and the same TV drama can be presented on the stage live, and that can also work for people without TVs, especially in informal settlements because we cannot say diabetes affects well-off people only [M, 40–49, Businessman, Buruburu]*

A few interviewees from Mukuru felt that delivering diabetes education sessions through existing local government structures, such as *barazas* (community meetings called by local chiefs) or *nyumba kumi* (community policing networks) would be useful to reach more people.

#### Final theory of change for diabetes prevention

**Fig 1:**
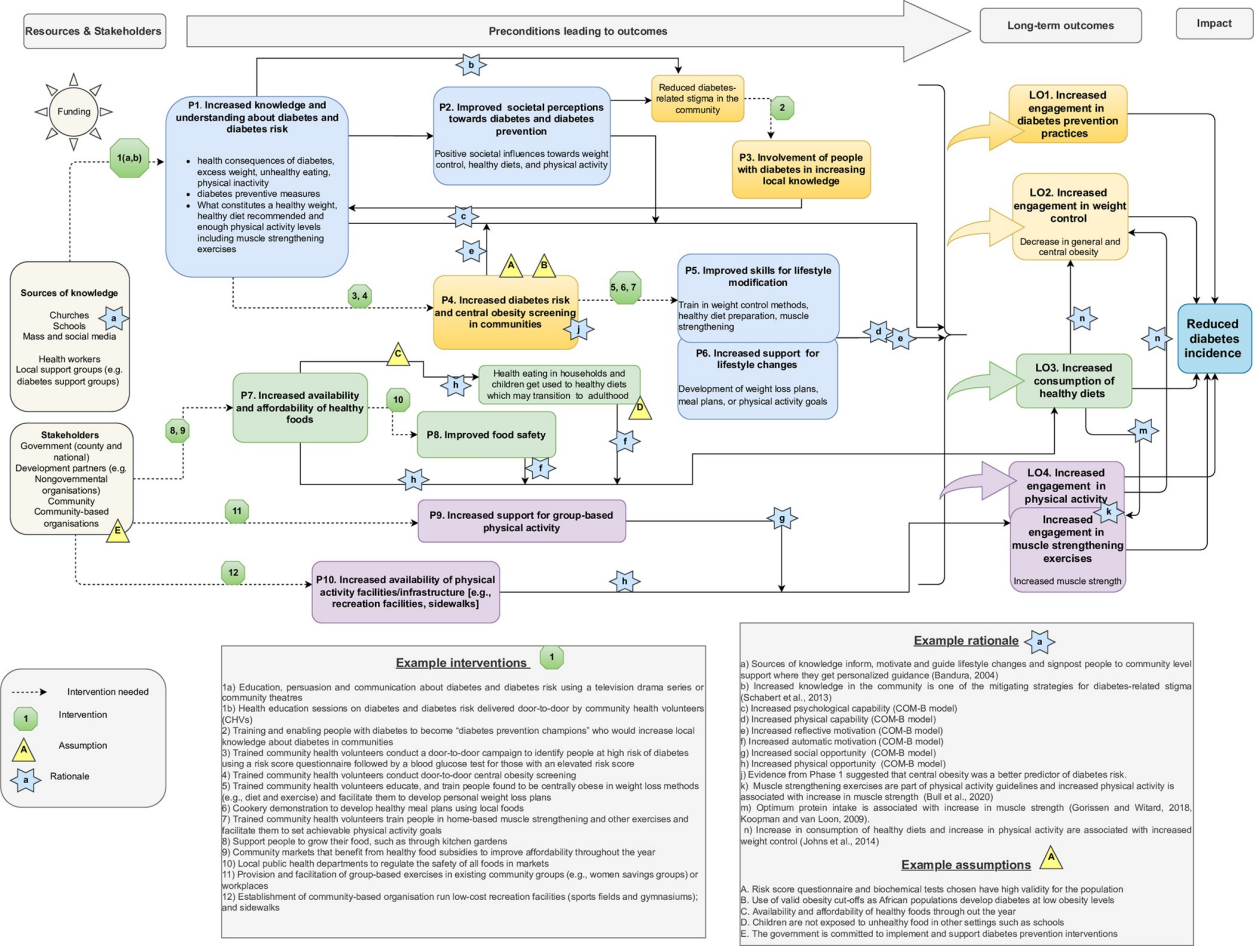
The theory of change for diabetes prevention in Nairobi, Kenya. Preconditions (P1-P10); Long-term outcomes (LO1, LO2, LO3, LO4); Interventions (1–12); Rationale (a-p); Assumptions (A, B, C, D, E).

As shown in Fig 1, based on the residents’ feedback, Intervention 1 (television drama) was revised to include community theatre and a community health volunteer door-to-door campaign. Additionally, another assumption, namely government commitment to implementing diabetes prevention interventions, was added.

Potential interactions between the long-term outcomes were conceptualised based on the scientific evidence. As shown in Fig 1, there is a bidirectional relationship between muscle strength and physical activity [38] (Rationale k in Fig 1). Also, increased eating of healthy diets, especially optimum protein intake, is associated with an increase in muscle strength [39] (Rationale m). Finally, increased eating of healthy diets and increased engagement in physical activity are associated with weight control [40] (Rationale n).

## Discussion

We used in-depth interviews and a co-creation approach, guided by the Behaviour Change Wheel framework, to develop a context specific theory of change for diabetes prevention in low and middle-income communities in Nairobi, Kenya. The in-depth interviews found the following key factors as influencing diabetes preventive practices in both communities: knowledge and skills for diabetes prevention, understanding of the benefits/consequences of (un)healthy lifestyle, social influences (e.g., upbringing, societal perceptions), and environmental contexts (e.g., access to (un)healthy foods and physical activity facilities). Gaps in these factors were particularly common in the low-income community such as low knowledge and understanding of diabetes risk; and limited access to physical environments to support healthy lifestyles e.g., poor active travel infrastructure and unaffordability of healthy foods. The proposed interventions for diabetes prevention included: increasing knowledge and understanding of diabetes risk and preventive measures (particularly in the low-income community); supporting lifestyle modification (i.e., goal setting and action planning); identifying people at high risk of diabetes through screening; and creating social and physical environments that support healthy lifestyles (particularly in the low-income community). Use of a co-creation approach allowed community participation in intervention selection, including ensuring that interventions do not create or increase health inequalities [41]. Community residents agreed the identified interventions were broadly feasible for diabetes prevention in Nairobi, but proposed community health volunteer door-to door campaigns and community theatre as strategies to spread knowledge. Those in the lower-income community in particular had concerns of government’s lack of prioritisation in implementing population-level interventions such as access to healthy foods and physical activity facilities.

We found that interventions that target individual-level capability and motivation could be important for diabetes prevention in both communities. These included increasing knowledge and understanding about diabetes risk, which were more relevant in the low-income community although knowledge gaps on what constitutes a healthy weight or a healthy diet, or enough physical activity were in both communities. Further, both communities would benefit from interventions to improve skills and action planning for lifestyle modification. This is consistent with evidence of a systematic review, of randomised controlled trials from high-income countries, which found that education, counselling, action planning, and upskilling were some of the behavioural strategies used in interventions that reduced the incidence of diabetes [42]. Current diabetes prevention strategies in Kenya focus on increasing knowledge through providing information, education, and communication materials in public health facilities, and media campaigns, primarily during World Diabetes Day [16] which may not be optimal: general knowledge is not enough for behaviour change. There is therefore a need for structured health education on diabetes risk and prevention similar to what is offered in the UK (person-centred, ongoing education using various learning styles) with an aim of developing knowledge, skills, and positive beliefs and attitudes towards lifestyle modification for those at high risk [43].

Apart from individual-level interventions, our study demonstrated the need for supportive social (community-level) and physical (environmental and cost related) interventions for diabetes prevention. These were particularly relevant to the low-income community and included creating positive societal attitudes towards healthy living; increased access to healthy foods and physical activity facilities; and improved active travel infrastructure. Nevertheless, an intervention that promoted group-based physical activity (women’s football) was already in existence in the low-income community which could be built on and expanded. Our findings are supported by a prospective USA study which found that modifying the environment to increase access to healthy foods and physical activity facilities was associated with a lower diabetes incidence [44]. Furthermore, the Kerala Diabetes Prevention Program in India, which led to a nonsignificant reduction in diabetes incidence, used strategies to modify the social and physical environment such as establishing kitchen gardens and walking groups in addition to individual level interventions [45]. Implementation of some the proposed environmental-level interventions, such as access to healthy foods and physical activity infrastructure, would require government involvement. However, some participants particularly from the low-income community expressed doubts about the government’s commitment to implementing such interventions. Therefore, there is need for increased political will to implement such interventions and future studies could investigate strategies that could be effective to increase political will.

Finally, this study found differences, in practices, barriers and facilitators of lifestyle modification, between the two communities. Particularly, the low-income community had lower knowledge, understanding of diabetes risk and access to environmental opportunities that enable healthy living (access to healthy food, physical activity facilities and infrastructure) compared to the middle-income community. This finding is consistent with a quantitative study in Ethiopia which found that low-income was associated with low diabetes knowledge level [46]. Given that residents in the low-income community reported an increasing diabetes prevalence, there is an urgent need for both individual-level (increasing knowledge) and population-level (increasing access to services, healthy foods, physical activity facilities) interventions to prevent diabetes in low-income communities.

### Strengths and limitations

The strengths of this study are recruitment of residents from two socioeconomically distinct communities making the findings relevant to a vast majority of the Nairobi population. Additionally, use of the Behaviour Change Wheel and Behaviour Change Techniques to develop a theory of change which was then reviewed by community residents contributes to the robustness of findings. It adds to the limited local evidence on diabetes risk perceptions and interventions for diabetes prevention. Nevertheless, our study had some limitations which should be considered in utility of the findings. First, the fact that COVID-19 restrictions meant the author chose the intervention examples and was unable to co-create the theory of change with local residents (as had originally been planned) may have reduced context-specific input and community ownership [29, 47, 48]. However, to mitigate this, the author mostly drew on information (including examples of interventions) provided during the in-depth interviews to inform the choice of intervention examples. Additionally, the author presented the developed theory of change to a subset of participants who took part in the in-depth interviews for quality review and input. Nevertheless, use of in-depth interview findings and quality review and input by a subset of participants may limit the transferability of the views gathered.

## Conclusion

Diabetes prevention interventions in Kenya should target various influences on behaviour at different levels. Increasing knowledge and understanding of diabetes risk, including what constitutes a healthy weight, (un)healthy diet, and sufficient physical activity, may increase the capability and motivation for lifestyle changes. Furthermore, increasing access to supportive social (community-level) and physical (environmental and cost related) interventions could contribute to healthy living. These interventions are particularly needed in low-income communities where there was low knowledge and understanding of diabetes risk and limited access to physical environments supporting healthy living such as poor active travel infrastructure and unaffordability of healthy foods. Finally, there is need for more political will for the implementation of these interventions and meaningful community participation during intervention development.

## Supporting information

S1 Questionnaire Inclusivity in global research questionnaire. (DOCX)

S1 File In-depth interview topic guide and Table 1. (DOCX)

S2 File Transcript excerpts. (DOCX)

S1 Fig (ZIP)
