# Supplementary material for: Perceptions of diabetes risk and prevention in Nairobi, Kenya: A qualitative and theory of change development study
Source: PLoS One. 2024 Feb 13;19(2):e0297779. doi: 10.1371/journal.pone.0297779 (PMC10863861; doi:10.1371/journal.pone.0297779)
Supplement: S1 File — (DOCX) [file pone.0297779.s002.docx]

**S1 Appendix**

# S1 Text: In-depth interview topic guide

**Introduction**

Firstly, I would like to say thank you for taking the time to speak to me about your experience of diabetes risk as resident of Nairobi. This interview will take around one hour. I would just like to confirm that you have provided full consent to participate in this research and you have the right to withdraw at any time. Can I just check that you are still happy to go ahead with this interview?

(If respondent agrees). [Read out full consent] In order for us to keep a good record of people’s views we would like to audio record this interview. Please can you confirm that this is OK for you? (If respondent agrees). Thank you. The audio-recording of the interview will be transcribed verbatim and digital copies will be stored securely for up to 10 years according to the University of Glasgow guidelines. Please feel free to speak your mind in the interview and be as honest as possible in response to each of the questions. I am keen to hear about your perceptions, perspectives, experiences and those of people in your community regarding diabetes risk and lifestyles. Everything you say will be kept in strictest confidence. If there are any questions you don’t want to answer for any reason we can just move on to the next. Is this all ok with you?

**In-depth interview details**

| **Date of interview:** | **Interviewer:** |
| --- | --- |
| **Venue:** | **Study site:** |
| **Time start:** | **Time stop:** |

**Participant demographic data capture**

I will ask you some basic details about yourself. These details will not be shared with anyone outside the research team.

| **Age** |  |
| --- | --- |
| **Gender** |  |
| **Occupation** |  |
| **Highest education level** |  |

**Community health and diabetes**

1. So, as you know this interview is about people’s health and in particular the health of people in your community. Can you tell me about the health problems that people here experience?
2. Can you rank for me the health problems you have mentioned starting with the one you consider most? *Prompt on why diabetes ranks there; why*
3. Do you know people with diabetes in your community? *Prompt on how they know them; prompt on why they don’t know people with diabetes – low disclosure? Stigma?*
4. What do you/people in your community think causes diabetes? *Prompt for all causes and risk factors – and for each ask how they know this*
5. What do you understand by family history of diabetes? Is this common in your community? In your opinion, do you think diabetes can be prevented if one has a family history?
6. Now can you tell me about anything that is already happening in your community that is being done to prevent diabetes.

Okay, so scientific evidence is now suggesting that some of the main risk factors for diabetes are being overweight, having a poor diet and being physically inactive, and I now want to look at each of these in turn

**Weight**

1. Would you say being overweight is a problem in your community?

If yes, why do you think this is? *Prompt, if not mentioned, on diet types, physical inactivity, hereditary or Gods will, contraceptives in women, childbirth*

1. What are the community perceptions on weight loss? Have these changed over time?
2. What would prevent people from losing weight? What would help them? *Prompt on personal, networks, settings, sector and cultural factors*

**Diet**

1. What are the most common foods and drinks you take in your family? Why do you take these foods often?
2. How do you prepare most of your food and beverages? *Prompt on boiling, frying, eating raw, adding sugar in beverages.*
3. How often do you/people in your community take foods high in sugar such as sweetened beverages, juice concentrate, cakes and biscuit? *Prompt on why this is the case*
4. In your opinion, what constitutes a healthy diet? *Prompt on recommended servings of fruit and vegetables*

- Are you/family/people in your community able to take what you consider a healthy diet frequently? How do you do this? If no, why not?
- Which components of a healthy diet are more common in diets and which ones are rare? *Prompt on intake fruits and vegetables*
- What stops or makes it difficult for you/your family/people in your community to take a healthy diet more frequently? *Prompt on affordability, availability, lack of knowledge*

1. What do you think constitutes an unhealthy diet?

- How frequent do you/your family take unhealthy diets?
- Which unhealthy diets or components of unhealthy diets are most common?
- Why do you/people take unhealthy diets or what makes it easier for them to take these diets more frequently? *Prompt on affordability, availability, lack of knowledge*

1. How could we support people to change the way they eat? *Prompt on personal, networks, settings, sector and cultural factors*

**Physical activity**

1. What do you understand by physical activity?
   - How do people perceive physical activity*? Prompt on stigmatised, accepted, viewed as a weight loss strategy only or not part of African culture*
   - Have perceptions of physical activity changed if you compare with 10 years ago for example? Why is this?
2. What forms of physical activity do you take part in? What of your people in your community what are most common forms of physical activity they take part in?
3. How do people in your community perceive walking? Why is this?

- What do you think stops or makes it difficult for people to walk?
- How do you think walking can be increased in the community? (What would motivate people to walk?)

1. What motivates you/people in your community to be physically active? Which are the most important motivations? Why?
2. What stops or makes it difficult for people to exercise? How do you think these barriers can be overcome?
3. How would people become more physically active in their daily routines?

- Do you know the health recommended amount of physical activity in a week? If yes, tell me about it. What was your source of this information?

**S1 Table 1: Effectiveness of intervention examples used in Table 4 in other settings or diseases.**

| **Precondition** | **Intervention example** | **Evidence to support effectiveness of intervention example** |
| --- | --- | --- |
| Preconditions 1 and 2 | 1. A television drama programme to increase knowledge and understanding about personal diabetes risk, weight, diet, and physical activity, and signpost people to other sources of knowledge, such as health workers, for more information. | - Television and radio drama programmes improved use of family planning services in Kenya and knowledge and understanding of HIV risk and preventive measures in Tanzania [1] - Intervention strategy based on the rationale that the media can be used to create a dual path of influence, i.e., to encourage behaviour change directly and to signpost people to relevant sources of information [2] - Increase in diabetes-related knowledge may lead to reduced diabetes-related stigma (important for Precondition 3) more knowledge was associated with lower levels of stigma in Kenya [3] |
| Precondition 3 | 1. Training and enabling people with diabetes to become “diabetes prevention champions” who would increase local knowledge about diabetes in communities. | - Disclosure by people with HIV/AIDS led to eagerness to know about prevention among uninfected SSA migrant women [4] |
| Precondition 4 | 1. Trained community health volunteers conduct a door-to-door campaign to identify people at high risk of diabetes using a risk score questionnaire followed by a blood glucose test for those with an elevated risk score. 2. Trained community health volunteers could conduct door-to-door central obesity screening. | - Door-to-door campaigns successfully delivered diabetes and hypertension screening interventions in Kenya [5] - Community health worker-administered diabetes risk score questionnaires and referral of those identified to be at an elevated risk for blood tests at a health facility has successfully been used for risk stratification in a recent pilot of a Diabetes Prevention Program in South Africa [6] - Screening for central obesity is informed by its higher predictive ability of diabetes risk than general obesity (measured using BMI) in this population[7] |
| Preconditions 5 and 6 | 1. Trained community health volunteers educate, and train people found to be centrally obese in weight loss methods (e.g., diet and exercise) and facilitate them to develop personal weight loss plans. 2. Cookery demonstration to develop healthy meal plans using local foods. 3. Trained community health volunteers train people in home-based muscle strengthening and other exercises and facilitate them to set achievable physical activity goals. | - Observational learning (e.g., cooking demonstrations), goal setting, and action planning were some of the intervention components that facilitated lifestyle changes that lowered diabetes incidence in high-income countries[8, 9]. - Inclusion of muscle-strengthening exercises based on evidence that diabetes is associated with low muscle strength in this population[7]. |
| Precondition 7 | 1. Support people to grow their food, such as through kitchen gardens | - Kerala Diabetes Prevention Program in India, which encouraged participants to establish kitchen gardens [10] |
| Precondition 7 | 1. Community markets that benefit from healthy food subsidies to improve affordability throughout the year | - Health Program in South Africa, an intervention offering discounts on selected foods to over 250,000 households, was associated with a higher intake of fruit, vegetables, and whole grains and a lower intake of foods high in sugar and saturated fat [11] |
| Precondition 8 | 1. Local public health departments to regulate the safety of all foods in markets | - Study from Taiwan found that perceptions around food safety influenced how people perceived health benefits from foods, which consequently influenced food intake [12] |
| Precondition 9 | 1. Provision and facilitation of group-based exercises in existing community groups (e.g., women savings groups) or workplaces | - The Kerala Diabetes Prevention Program, a low-cost community-based lifestyle intervention in India that led to a non-significant decrease in new cases of diabetes, used yoga training and walking groups to increase physical activity [10] |
| Precondition 10 | 1. Community-based organisation run low-cost recreation facilities (sports fields and gymnasiums); and sidewalks | - Free access to physical activity facilities and new transport infrastructure were associated with increased leisure physical activity [13] and active commuting [14] in the UK - Local marketing of the facilities through a community-based organisation was found to promote access and engagement in the UK [13] |
| Preconditions: 1) Increased knowledge and understanding about diabetes and diabetes risk; 2) Improved societal perceptions towards diabetes and diabetes risk; 3) Involvement of people with diabetes in increasing local knowledge; 4) Increased diabetes and central obesity screening in communities;5) Improved skills for lifestyle modification; 6) Increased support for lifestyle changes (i.e., weight control, healthy eating, physical activity); 7) Increased availability and affordability of healthy foods; 8) Improved food safety; 9) Increased support for group-based physical activity; 10) Increased availability and affordability of physical activity facilities and infrastructure | | |

**References**

1. Ryerson WN. The effectiveness of entertainment-education: Case studies from around the world. Using the media to achieve reproductive health and gender equity. 2011:72.

2. Bandura A. Health promotion by social cognitive means. Health education & behavior. 2004;31(2):143-64.

3. Hamra M, Ross MW, Orrs M, D'Agostino A. Relationship between expressed HIV/AIDS-related stigma and HIV-beliefs/knowledge and behaviour in families of HIV infected children in Kenya. Tropical medicine & international health : TM & IH. 2006;11(4):513-27. Epub 2006/03/24. doi: 10.1111/j.1365-3156.2006.01583.x. PubMed PMID: 16553935.

4. Arrey AE, Bilsen J, Lacor P, Deschepper R. “It’s my secret”: fear of disclosure among Sub-Saharan African migrant women living with HIV/AIDS in Belgium. PLoS One. 2015;10(3):e0119653.

5. Pastakia SD, Ali SM, Kamano JH, Akwanalo CO, Ndege SK, Buckwalter VL, et al. Screening for diabetes and hypertension in a rural low income setting in western Kenya utilizing home-based and community-based strategies. Globalization and Health. 2013;9(1):21. doi: 10.1186/1744-8603-9-21.

6. Hill J, Peer N, Jonathan D, Mayige M, Sobngwi E, Kengne AP. Findings from Community-Based Screenings for Type 2 Diabetes Mellitus in at Risk Communities in Cape Town, South Africa: A Pilot Study. International journal of environmental research and public health. 2020;17(8):2876. doi: 10.3390/ijerph17082876. PubMed PMID: 32326364.

7. Manyara AM, Mwaniki E, Gray CM, Gill JM. Comparison of risk factors between people with type 2 diabetes and matched controls in Nairobi, Kenya. Tropical Medicine & International Health. 2021;26(9):1075-87.

8. Baker MK, Simpson K, Lloyd B, Bauman AE, Singh MA. Behavioral strategies in diabetes prevention programs: a systematic review of randomized controlled trials. Diabetes Res Clin Pract. 2011;91(1):1-12. Epub 2010/07/27. doi: 10.1016/j.diabres.2010.06.030. PubMed PMID: 20655610.

9. Cradock KA, ÓLaighin G, Finucane FM, Gainforth HL, Quinlan LR, Ginis KAM. Behaviour change techniques targeting both diet and physical activity in type 2 diabetes: A systematic review and meta-analysis. International Journal of Behavioral Nutrition and Physical Activity. 2017;14(1):18. doi: 10.1186/s12966-016-0436-0.

10. Thankappan KR, Sathish T, Tapp RJ, Shaw JE, Lotfaliany M, Wolfe R, et al. A peer-support lifestyle intervention for preventing type 2 diabetes in India: A cluster-randomized controlled trial of the Kerala Diabetes Prevention Program. PLoS Med. 2018;15(6):e1002575-e. doi: 10.1371/journal.pmed.1002575. PubMed PMID: 29874236.

11. An R, Patel D, Segal D, Sturm R. Eating better for less: a national discount program for healthy food purchases in South Africa. Am J Health Behav. 2013;37(1):56-61. doi: 10.5993/AJHB.37.1.6. PubMed PMID: 22943101.

12. Wang ES-T, Tsai M-C. Effects of the perception of traceable fresh food safety and nutrition on perceived health benefits, affective commitment, and repurchase intention. Food Quality and Preference. 2019;78:103723. doi: <https://doi.org/10.1016/j.foodqual.2019.103723>.

13. Higgerson J, Halliday E, Ortiz-Nunez A, Brown R, Barr B. Impact of free access to leisure facilities and community outreach on inequalities in physical activity: a quasi-experimental study. J Epidemiol Community Health. 2018;72(3):252-8. doi: 10.1136/jech-2017-209882.

14. Panter J, Heinen E, Mackett R, Ogilvie D. Impact of New Transport Infrastructure on Walking, Cycling, and Physical Activity. American Journal of Preventive Medicine. 2016;50(2):e45-e53. doi: <https://doi.org/10.1016/j.amepre.2015.09.021>.
