# Supplementary material for: Perceptions of diabetes risk and prevention in Nairobi, Kenya: A qualitative and theory of change development study
Source: PLoS One. 2024 Feb 13;19(2):e0297779. doi: 10.1371/journal.pone.0297779 (PMC10863861; doi:10.1371/journal.pone.0297779)
Supplement: S2 File — (DOCX) [file pone.0297779.s003.docx]

|  | **Excerpts of the transcripts** |
| --- | --- |
| 1 | R: if I am passing somewhere,  M: mmm  R: and I find them at some place, I can buy them if I have the money.  M: mhm.  R: eeh.  M: why are you not consuming them often?  R: because, you know the availability is not easy.  M: finding them in the market?  R: yes, our market which is here.  M: okay.  R: and again what they bring here is not good, they just bring the bad ones.  M: mmm  R: at times they bring the waste, the ones that have been thrown away; they collect that and  bring to us.  M: mmm  R: you know what happens, in the market there are things like cabbage and so many things  that have been dumped there. The people from that area go and collect all that, put it in  one place then they start selling that. Now, you find that our sellers from this place go  collect that and sell to us. You will find this being sold at a cheap price because they also  bought it cheap.  M: mmm  R: for example the avocado that you see for export, there are those that I hear that they get  thrown out there, near the airport. The people from this place, you see the village is near  there; they go there, collect that and bring to us. There are people who know that, that is  bad but with some, they will look at it, “this is an avocado?”, then they just take that.  M: mmm  R: they do not know that, if you look at it keenly, you will find that it has germs.  M: mmm, okay. And, aah, do you know the importance of fruits in the body? Are  fruits having any importance? For example in a day, are you supposed to eat fruits?  R: mmm  M: are you supposed to eat fruits or it is okay not to eat fruits?  R: I do try to eat fruits.  M: mmm  R: eeh, I like eating a fruit a day.  M: mmm  R: eeh, like now I have eaten one.  M: do you know you are supposed to eat how many fruits in a day?  R: I don’t know,  M: the approximation,  R: I do not know but that...that is what I can manage.  M: mmm  R: I can’t eat more than that.  M: that is what you can,  R: manage to consume because of finances. |
| 2 | M: what is the people’s view on this?  R: when a person is big, and they reduce the weight, there are people who say, you know the  people’s opinion is different, if a person felt that they are too big they might exercise to  reduce that much weight. There are people who might lose weight and the people will  start saying, “this person is? Is sickly.” But, they do not understand that the owner of the  body made the decision to reduce weight after realizing that they are overweight. But if  a person doesn’t know of this, they will start talking about things that this person is not  suffering from.  M: Okay  R: mmm  M: so, they will think that this person has a problem?  R: yes, there is a particular problem with their body. “Wasn’t this person big the other day  and now they are thin, could it be that this person is sick, isn’t this person sick? If this  persons health is like this maybe there is an underlying issue” but you might find that this  person has been exercising so that they can reduce that weight but people are just  contributing to different opinions. Mmm.  M: okay, do you think that people’s thoughts and opinions have changed over the years  when it comes to reducing weight? Maybe people in the village have started or not  started seeing the importance of weight reduction?  R: it has not. Like now there is a woman who wakes up and stands at the door, she has  constructed by the roadside, she just stands at the door, waits for a motorbike and sits on  it. With that motorbike, she will go for a very short distance where the people play ball.  She will alight there.  mmm, with that one, let’s say with that question you have asked me, for her she doesn’t  see the importance of walking, she is used to doing that. She has the money and she  doesn’t want to walk, she just feels fine with her body weight. But with her, it is even  difficult for her to walk. Mmm. |
| 3 | M: do you think that is a problem here in Mukuru?  R: ah-ah! It is not a problem in this place. Do you know, well, let me just tell you the truth.  Okay you know that with the body, mainly it will grow depending on what people eat.  Isn’t it?  M: mmm  R: there are foods that make you grow and get to be like that. So, to me, well, to me, what I  know is that most people inside here, most of them are low income people. You see?  They are not high class people. You will find that most people here, let’s say around, I  can’t say more than 55%, they normally, some of them they don’t even take lunch, they  only take supper. So, but here we do have the ones who are heavy, they get a big body,  very few of them, very few, very few.  M: okay, and in your opinion what causes that body weight, being fat with a lot of body  weight? What causes it? What causes that?  R: okay, first of all, you know there are some people who were born like that. They have  inherited that, the big body. That is the first point. Secondly, with the big body, I can’t  say if it is because of feeding well or what is the reason behind that. There I cannot tell  you, eeh. But you know, you can be born and inherit that from your parent. Your family  can be of big bodied people. You can get to become big bodied.  and there is a girl that I know, the parents do not have a big body but when you look at  her body, she is this size.  M: and apart from inheritance, is there any other cause for being very heavy weight?  R: being heavy weight?  M: mmm?  R: with that one I can’t lie. {Brief interruption} We can just proceed. |
| 4 | M: okay. There is the time that is deemed fit for a person to exercise in a week, do you  know how many minutes this could be?  R: the required time?  M: the required amount of time that a person is supposed to exercise in a week?  R: there is a person...there is the time each person can be able to exercise. For my case, I  stopped playing ‘ball’ a while back but I can exercise for one hour. You see? I can  decide to exercise for one hour, when that one hour gets finished, that will be it. I will  stop exercising, go and take a bath then relax. There is a person, who can’t take that  long, but the important thing with exercising, when you do your usual exercising, you are  supposed to exercise for 1 hour and 20 minutes.  M: is that in a week or in a day?  R: in a day, every day, it should be 1 hour and 20 minutes. With that, you will have  exercised but if you are really determined, you are supposed to exercise for 2 hours 40  minutes. You see? That is 1 hour 20 minutes in the morning and 1 hour 20 minutes in  the evening.  M: okay.  R: that is in a day, you make sure that in a day, you exercise for 2 hours 40 minutes every  day.  M: how did you get the information that you are supposed to practice for 1 hour 20  minutes?  R: (no) with that one, it doesn’t mean that I was given that information but that is what I am  used to doing when I was alone, I ensured that if I am not playing ‘ball’, then I would do  that. this is because, with that timing I can go up the hill, I can do some different kinds of  exercising and there is a way the body recovers, you know when you are at some place  and an object falls, you have to move out of the way, but then, you can’t move out of the  way because of lack of exercise. That moving out of the way from a falling object is  also some sort of exercise, if a wall is falling you need to jump out of the way, if a tree is  falling, you should jump out of its way, or if a tree is falling, you need to get out of its  way.  M: what gives people motivation to exercise?  R: aah, this one, this one is tricky. With this place even...  M: = even the ones...=  R: =even the youths = even with the youths, you can mention to a person something to do  with exercising and they will tell you, “Why do I need to exercise? That is not my thing,  I do not want to do that. With exercise I will go looking for hunger and when I come  back I will feel like eating”. You see? Many of them talk about that.  M: even the ones who are obese?  R: with the ones who are obese, like I said, you have to give them an incentive for them to  do that so that they can exercise.  M: okay.  R: and again you have to have stayed with them for a long time so that you speak with them  about that. But again, I feel that those obese ones will not refuse if at all you properly  explain this to them.  M: okay, is there anything that we have not mentioned that is preventing the people  from exercising and maybe, if that is done away with, many people will start  exercising?  R: something that can prevent this is when you find a youth who is still young and they used  to play, they used to exercise, but now it has reached a point...you know there is that life  where they were sleeping and eating at home but now he has to cater for his food. This is  what we refer to as ‘Harambee’ you see? It reaches a point that at home, this youth is no  longer a child and theya re starting their race and now this depends on how quick he is.  At work, maybe he has gotten a job in a company and the time when he would have been  exercising is the time that he now goes to work and probably he is on night shift. When  eh leaves work, it will be morning and then it means he has to go and sleep, with that,  you see he will not be able to exercise?  M: mmm  R: you see?  M: okay.  R: so, this will be affected by the income and many youths will be affected because of the  kind of work that they do.  M: what of the old people?  R: with these ones they have already done away with exercising. They are playing the ‘final  game’. Mmm, you know with an old person, maybe they trained when they were young,  M: now they are old,  R: they trained during their time and if at all they have to do it in their old age, not unless  they are not going to work, with that, maybe you can tell them, “Ha!, let’s go and  exercise”, with exercise it is important for the joints. You know when you age, if you are  idle, there is going to be stiffness in the body and this can creep in even when you are  young. With me, the kind of exercising that I used to do, if right now I just take 2 weeks,  not working but feeding, I can eat but still feel hungry. You see? This is because there is  something that is missing in my body.  if I feed and stay idle, I will feel hungry and my body will feel tired. When I get up, I  have to get up in a certain way because of that disability that comes with lack of  exercising and the kind of activities that you are used to doing. You can find a man has  aged and they can’t even walk because of the kind of activity that he was used to doing.  That goes hand in hand with exercise and for sure you are getting old but if you are still  walking, you will not start bending in any part of your body. But now, if you say that  you have aged and you are waiting for each child to send to you an Mpesa message, with  that one now, it is a must that you will get a challenge. |
| 5 | M: okay, apart from losing appetite what else makes you to feed on food that is  unhealthy?  R: eeh?  M: what makes you not to manage to eat healthy food every day?  R: you feed on food that you know that you will be fully satisfied. Eeh, but when you eat  that food today and then tomorrow you feed on the same, you will not be satisfied.  M: okay.  R: until you change it. mmm.  M: in your opinion what food is not healthy or in your opinion what food do you think  is unhealthy?  R: it doesn’t have a lot of health. I think it is meat, I think eating meat each and every day is  a problem. Eeh. Because,  M: okay, and apart from the meat...you were saying ‘because...’?  R: eeh?  M: why were you saying that eating meat every day is unhealthy?  R: my father in law liked to eat roasted meat all the time and now his legs swell. Mmm, he  now gets sick in the legs and they get swollen. Mmm.  M: apart from the meat, what other food do you think is unhealthy?  R: the one, the one that can make you to be healthy?  M: mmm?  R: also, even feeding on ugali every day, you need to keep alternating.  M: you need to alternate. In your opinion, how can people be assisted in feeding on  healthy food?  R: to help the people?  M: what can be done  R: eeh  M: so that people can feed on food that is healthy?  R: I think it is just like the way people go to the hospitals, when you go to the hospital,  maybe the clinics, the parents and mainly the women are the people who do the cooking.  They do cook the food. So, you will explain to them how to cook and how they can  change the foods from which food to which food. Eeh.  M: is that something that is happening in your hospital?  R: eeh in this area, the clinic has many children and these are young children. Eeh.  M: so they do explain to you on how to cook and the healthy foods and things like that?  R: eeh.  M: okay.  R: mmm  M: apart from education, what else needs to be done  R: eeh, the education.  M: so that people can feed on healthy food?  R: you just explain to them you announce to them and even when they go to the hospitals if  a disease is noted they can be advised on the same. Eeh.  M: okay, fine. Foods with protein, do you know of the foods that can give you protein?  R: eeh, I know.  M: what are the foods that you consume?  R: the beans.  M: beans?  R: beans, eeh.  M: how many times in a week do you manage to feed on food with proteins?  R: it can be twice.  M: twice?  R: twice or thrice.  M: in a week? |
| 6 | M: okay, apart from losing appetite what else makes you to feed on food that is  unhealthy?  R: eeh?  M: what makes you not to manage to eat healthy food every day?  R: you feed on food that you know that you will be fully satisfied. Eeh, but when you eat  that food today and then tomorrow you feed on the same, you will not be satisfied.  M: okay.  R: until you change it. mmm.  M: in your opinion what food is not healthy or in your opinion what food do you think  is unhealthy?  R: it doesn’t have a lot of health. I think it is meat, I think eating meat each and every day is  a problem. Eeh. Because,  M: okay, and apart from the meat...you were saying ‘because...’?  R: eeh?  M: why were you saying that eating meat every day is unhealthy?  R: my father in law liked to eat roasted meat all the time and now his legs swell. Mmm, he  now gets sick in the legs and they get swollen. Mmm.  M: apart from the meat, what other food do you think is unhealthy?  R: the one, the one that can make you to be healthy?  M: mmm?  R: also, even feeding on ugali every day, you need to keep alternating.  M: you need to alternate. In your opinion, how can people be assisted in feeding on  healthy food?  R: to help the people?  M: what can be done  R: eeh  M: so that people can feed on food that is healthy?  R: I think it is just like the way people go to the hospitals, when you go to the hospital,  maybe the clinics, the parents and mainly the women are the people who do the cooking.  They do cook the food. So, you will explain to them how to cook and how they can  change the foods from which food to which food. Eeh.  M: is that something that is happening in your hospital?  R: eeh in this area, the clinic has many children and these are young children. Eeh.  M: so they do explain to you on how to cook and the healthy foods and things like that?  R: eeh.  M: okay.  R: mmm  M: apart from education, what else needs to be done  R: eeh, the education.  M: so that people can feed on healthy food?  R: you just explain to them you announce to them and even when they go to the hospitals if  a disease is noted they can be advised on the same. Eeh.  M: okay, fine. Foods with protein, do you know of the foods that can give you protein?  R: eeh, I know.  M: what are the foods that you consume?  R: the beans.  M: beans?  R: beans, eeh.  M: how many times in a week do you manage to feed on food with proteins?  R: it can be twice.  M: twice?  R: twice or thrice.  M: in a week? |
| 7 | R: there is weight that you get born with, this one is god given. But, there is the one that  bring on yourself; you can get this one because of the way you feed. Eeh.  M: okay.  R: mmm  M: what of the family planning medication, do you think family planning can result to  body weight?  R: this one will not bring a lot of weight, it will be just a bit of weight, but you will find that  it will bring something like...blood pressure.  M: the family planning?  R: eeh.  M: is this something that you have witnessed?  R: I have seen this and I realized that I slightly had that problem eeh, there is a time I was  using it and it was not a big problem but before it got too much. I went to the doctor and  tried to find out where the problem is and the doctor told me to discontinue using that.  When I stopped, this made the problem to stop also.  M: the high blood pressure?  R: eeh. It stopped.  M: okay  R: mmm  M: apart from what we have discussed, what causes a lot of body weight?  R: with body weight, being too fat and being overweight?  M: mmm?  R: isn’t that because of the food?  M: okay.  R: or what is it? it is because of the food, or the being from a certain family or sometimes,  you can find a person doing a particular job, maybe in the gym. Sometimes you can  exercise and then the exercise ‘gets to like you’ {ikupende pia}  M: will exercise make you increase or add weight?  R: there are people who add weight and there are people who reduce weight. |
| 8 | M: okay. What do the people think of women who reduce weight? For example, when  you have a lot of weight then you reduce it, what will the people think or what  opinion will they have on this?  R: you know when you reduce, many diseases will not get you, but when you are heavy,  many illnesses will affect you. Mmm.  M: so is there an opinion that people normally have, this could be your friends or the  people in the village, what will they say or what opinion will they have?  R: people say so much when you reduce becaue they will say that maybe you have a  problem, sometimes they will say that you are sick, you know the way people think has a  lot of issues. Mmm. When you reduce they will say so many things, they will say that  this person is sick, they have aah this other disease. Eeh.  M: and with such thoughts or with what they say, can this make you not to reduce your  weight?  R: when you have...you can’t aah, you can’t stop them but you see, when you have too much  weight and you are the one who will be having a problem.  this will be too much for you, you will be overcome by illness and, getting a disease is  what will overcome you. So, if you look at people, you will not reduce weight. Eeh.  And again the doctor is the one who is telling that you should not be overweight becaue  of diabetes. Eeh.  M: apart from such talk, what else can make a person not to reduce weight? Maybe  they are obese but there is something that is making them not to lose weight, what  else can make a person not to reduce weight?  R: this is becaue people will think that you have this other illness, this one of AIDS eeh.  M: so they will think that you have it?  R: eeh. They will think that you have it if you lose weight. If you reduce weight, meaning  that if you are very thin. Eeh.  M: and apart from those thoughts, is there anything else in this village preventing the  people  R: mmm  M: from losing weight here in the village?  R: you know if people lose weight, we could not be getting diabetes. This is becaue diabetes  is caused by being overweight. Eeh.  M: okay, so what makes.  R: but my opinion is for the people to reduce, to reduce that weight. Eeh.  M: what can make a person not to be able to reduce weight?  R: why?  M: is there anything that can make people not to be able to reduce weight?  R: isn’t it because of those words where a person is being told, “you are sick” that is what  the people fear.  M: okay.  R: mmm  M: okay we can move to issues related with food now.  R: mmm  M: what is the food that you consume most often in your home?  R: we just use, when I get rice I cook it, when I get ugali, I cook it. This is becaue now you  know that our income is low. Mmm. |
| 9 | M: and, food that is healthy, or healthy food,  R: yeah  M: in your opinion, what does it comprise of?  R: healthy food?  M: yes  R: if it is rice, I can boil beans, I do not buy boiled beans from out there. so when I boil  these beans, I will cook stew, then we can eat that with the rice. Isn’t that healthy food?  Rice is white, and when you eat rice with no green in it, or if you eat the aah beans only,  then this food is not healthy.  potatoes are healthy and they add heat in the body. When you cook rice and beans, that is  food that adds heat in the body, they increase the body’s health. With Githeri, if you take  beans and maize and mix that, then you go and pick the leaves of pumpkins or the  traditional vegetables and then you prepare that for the children, do not fry it, cook it as it  is, even if you cook it like that and serve them with tea, with what?  M: with tea.  R: that is healthy food.  M: okay.  R: if not that, let’s come to ugali, go and pick your spinach and kales, cut the vegetable and  prepare ugali with that. They will be satisfied with that and that is healthy food.  It is not a matter of saying that the child is hungry then you take milk and Weetabix.  That will not be healthy for a child’s body. This is something with chemicals. We do not  even know how the milk has been milked. You do not also know how the Weetabix has  been prepared and then you feed the child on that. Won’t you be feeding the child on  chemicals?  M: mmm  R: the sweet potatoes, the potatoes, the matoke bananas, arrowroots, if you put a small  onion, corianders, and fry it for the children then you serve as it is, make a point of not  adding Blue Band on top.  M: okay.  R: Blue Band is full of chemicals and you need to feed your children on food that is?  M: healthy.  R: that is healthy. Most of the times you can say that your children will drink soup from  time to time. You can buy the bones from the butchery then you boil at home. You will  not cook tea in the morning. They will drink soup for breakfast in the morning. I will  not make tea during that day. First of all that is food that is what?  M: healthy.  R: so, I can say that is healthy food. At home, I have a week where I feed them on soup,  another week I feed them on porridge and there is a week where I prepare tea.  M: okay.  R: yeah.  M: you have talked of beans,  R: yeah.  M: and you have said that you boil it for yourself  R: yeah  M: and that you do not get this from out there. Why have you said that?  R: the beans you buy from out there, there are people who put chemicals in the beans. That  chemical they put is called magadi. There are people who will buy Panadol and dip it in  there so that it can cook quickly. There are people who take chemicals, some other  chemicals... they will dip it in the beans so that it can cook quickly. The more you eat  this, the more you get gas in your stomach and you start suffering from heart burn.  when you boil the food for yourself, you will not add those things, this is becaue you are  not doing that for business. The person who is adding those things is doing that for what?  M: for business.  R: they will start cooking at 8 and by 1 they will be selling the food that is raw cooked. I do  not like to buy those things that are sold out there because of the things that have those  additives. Yeah.  M: would you say that you feed on healthy food every day?  R: if I try within my income, if in my income I find that I have 200 shillings for that day, I  will try to feed on healthy food but if I do not have that, then we will eat what we are  used to eating. Yeah.  M: what else prevents you from feeding on healthy food apart from the income?  R: any other thing that is preventing me?  M: mhm.  R: there isn’t, you know those things cost money? Yeah.  M: okay. You have mentioned vegetables in the food. What of the fruits?  R: the fruits?  M: mmm.  R: haven’t I told you that at my home when we wake up in the morning a person can decide  to eat fruits. They can go and get a pineapple, water melon and then they can survive on  that till evening?  M: eeh.  R: yeah.  M: do you know the amount of vegetables and fruits  R: eeh  M: that a person is supposed to consume in a day?  R: I do not understand about that but we do eat that, a person eats that till they say that they  are full. Yeah.  M: okay.  R: yeah.  M: as we finish on the food,  R: yeah  M: what can happen in Mukuru  R: yeah  M: so that people can eat healthy food more?  R: you know a person eats... I told you very well, that if you have money, there is no way  you will not feed properly feed your family. If you don’t have, what will you use to buy  those things?  M: nothing.  R: the main contributor for that is lack of jobs, being jobless”?” The more you are jobless,  or you do casual work of washing clothes and you do not know if tomorrow you will  have any income, then you will definitely have a problem when feeding your family.  Yeah.  M: okay.  R: yeah. |
| 10 | M: what health problems do you see that are affecting the people in Buruburu?  R: mmm, I think the old people are the ones who have those complications, the mmm health  complications. The ones who have arthritis, the ones who have this problem with  ‘sugar’, that is diabetes, blood pressure, and aah maybe I think.. so far I think it is just  those oens.  M: okay. So the main problem in Buruburu is arthritis, diabetes, and high blood  pressure. Anything else?  R: mmm I think that could be it. This is because, you know with most of the people who  live in Buruburu are the people who bought houses long ago. They have old age, these  are old age people, these are the people who are developing those things, those  complications. Like my landlady is also having blood that pressure, the, the husband  who was, who was of the landlady, he also had complication with blood pressure and  then it turned to the kidneys and cancer and so on then he passed away. He passed away  last year. Aah I have also seen my parents with blood pressure. Mmm  M: they also live here in Buruburu?  R: yes, they are in Buruburu, mmm.  M: so, how do you rank the diseases you have mentioned, which one would you say is  the major one, that is affecting the people in Buruburu and where would you rank  diabetes?  R: mmm I, I don’t know whether I can generalize, I have just interacted with the people that  I have seen, I do not know about other people. But, I think so far, aam that diabetes, I  think it is because of the lifestyle of the people of Buruburu. I think it is just diabetes and  blood pressure. Mmm.  M: okay, do you know many people who have diabetes?  R: yeah, actually so many, not necessarily from Buruburu  M: yes  R: but I know so many.  M: what about in Buruburu do you know a number who have diabetes?  R: in Buruburu, I can name 10.  M: 10 in Buruburu?  R: mmm  M: okay.  R: mmm  M: you would say that it is a problem that is there?  R: it is prevalent here, mmm.  M: what do you think causes diabetes, in your opinion?  R: as far as my knowledge or I am concerned, I think it has to do with lifestyle. It has to do  with the lifestyle and maybe the food, the type of food we eat here. Yeah maybe there is  a person who was drinking a lot of beer.  you know taking a lot of alcohol also affects your, ‘I don’t know’, it affects your aah,  your heart, your heart beat and applies to your blood pressure, it creates a complication of  the blood pressure. And aah it is a fact that in here people like eat starchy-starchy  carbohydrates. People eat pizza, people eat chips, people take soda, a lot of soda, yeah.  M: okay  R: those are the two main causes. Other than that also, I think that the people are not doing  a lot of exercise. People just stay in the houses. They are either in the house or the  office, from the house, to the office. Yeah.  M: okay, so you have mentioned not doing exercise and diet.  R: mmm  M: and lifestyle. And by lifestyle what exactly do you mean?  R: lifestyle in terms of taking of alcohol. Buruburu is a centre party area, a party area. So  the only kind of recreation we have is going to the bar to party. Mmm. A lot of alcohol  is harmful to health also. |
| 11 | M: there is also a perception of, and this is for women and also for men, the African, it&#39;s  African to be big, big and beautiful and such kind of perception. Do you think they  may be contributing to people being overweight and maintaining?  R: tremendously, tremendously. It contributes. Once people think that when you are big,  now you are doing well in life, when you are, when you are huge, you know, when your  body is big, you are doing very, very well in life, I mean, now, society perceives to you  being wealthy.  but when you are lean or malnourished, you are not doing well. {Laughs} yeah? You can  be lean, you are quite healthy, but the perception in society is not going to look at you as,  “hey, you are a wealthy man?” that is the funny thing how, how the society perceives it  actually in Africa which is wrong. It is a misconception in Africa which is actually  wrong. Most, most people who are huge, they are more prone to diseases. Yeah?  M: yes  R: aah I am not saying that people who are lean are not prone, they are also prone but, if you  are overweight, definitely another thing that has affected, your organs, your vitals, you  know? Becaue of the amount of cholesterol in your body, blood pressure, diseases are  more, you are more prone to diseases than someone who is (lighter than you)  M: and is these perception in among your peers so, you are quite young, and also you  are well educated, is this a perception that you see among the people, your friends  and your peers?  R: it is. Actually in Buruburu it is. You know aah Buruburu primarily, there is accessibility  of alcohol. Drugs not that much but alcohol, aah cannabis smoking, cigarette smoking,  so in these kinds of engagement, and talking of aah foods rich in sugar, you know starch,  all these kinds of meaty, meaty foods sold that are being sold in fast foods and all that,  yeah?  M: mhm  R: Buruburu is always quite exposed becaue, my peers, my peers are quite exposed because  most of them drink, most of them want to get these kinds of foods, and, it is a major  health factor that aah risk that most of them, most of us actually we are more exposed to  diabetes as compared to...  M: older people. Older generations?  R: yes.  M: how do people perceive weight loss? So what are the community perceptions on  weight loss? So if you had some weight and you lost it, what are the perceptions of  your peers or the community?  R: you are sick, you are sick. That is the first thing. “Ha! so and so is sick. Did you see her?  I think she is sick. I think she is sick.” Anyway you have lost weight, you are not doing  well.  M: yeah.  R: there is something that is totally affecting you, weight wasting. That is the first  perception that comes to mind. Weight is wrong, weight is totally wrong. Weight is  actually seen as a totally healthy thing. becaue you will see every morning people want  to lose weight, especially ladies they want to start running. Ladies will do that to look  beautiful.  M: yes.  R: not the healthy part of it, they do that to look beautiful, have a sexy body, look nice, you  know? Or maybe {00:29:27} so every morning, or every evening running around Buruburu  going round it.  M: yes.  R: Buruburu to Jogoo Road and coming back, from phase 1 to phase 5. You know, just do  that every single day to lose weight, (despite the fact that it is for to look nice and to lose  weight).  M: yeah.  R: but you know the wrong thing is this, someone will do that, 2-3 days, 4 days, on the 5 th  day, going to KFC, there is KFC in Buruburu.  M: yes  R: there is Kiza {laughs} you know? You have lost all these calories then on top of it you are  adding one again. In the evening when eating, it is meat and pork and drinking. Do you  think it is really affecting anything?  M: it is zero game.  R: {laughs} to a great extent. That is a fact that most of, most of my peers are afraid of, eating.  That if it was traditional meals, yeah. Healthy foods, also appears a lot.  M: you have said that theya re afraid of eating healthy?  R: healthy food. Yeah, only fast foods, sodas junk food. Yeah.  M: okay, and the perceptions on weight loss, do you think they&#39;ve changed? So, if you  compare 10 years ago on how people perceived weight loss, do you think there has  been a change and improvement?  R: there is no improvement, it is actually getting worse. The, the, the, the more weight you  gain, the more your peers see as if you are doing well and the more you will also want to  proceed on that. Very few, very few individuals, that is very few individuals people like  you, usually know when ‘I should stop’.  in Swahili, they usually say, ‘I have provoked my body and it has shown me that it really  can’. So you will think, ‘let me reduce on taking KFC foods, let me reduce on taking  sodas, let me reduce on this.’ Why? Because at the end of the day I am getting a fat  belly, becaue even I have realized that, ‘hey, people are looking at me funny when doing  swimming, because I am trying to hide my belly and stuff like that. But generally, it is, it  is going, it is going worse, as you continue, because some of the times you feel it grow  fatter and fatter and fatter.  M: okay.  R: it is the lifestyle that you live, you know?  M: yeah  R: aah from work I will enter a matatus, from work I will get into the car, I will drive home,  enter Buruburu, from work I will go and sit in a bar, then drink slowly, get into the car,  get home and sleep.  M: yeah.  R: getting back, then you go and sleep, the next day is just the same. So it is growing worse  and worse, it is this thing with lifestyle. It is growing worse and worse. Because, there is  a ground for exercising  M: yes.  R: but there is a specific group of peers, who normally go there to work out. You know  because with them, you know, people are different, but the, the major percentage, we  tend to live that life of just relaxing, drinking. Guys in Buruburu like drinking, going  from work, you know. It will show that, it shows some affluence,. If to go playing  football, you know and your friends are going to drink. With the current society, ‘what  will someone work for? Let me go and drink and eat nyama choma.  M: yes  R: and my peers want to go and play football and do exercises for two hours, come back,  you are tired, that is your half day, then force you to go and drink.  M: yeah.  R: so it’s, it’s, it’s alcohol bit of it,  M: okay  R: it is the eeh eating bit of it,  M: yeah  R: junk and the lifestyle.  M: yes.  R: because most of your peers are doing that. |
| 12 | M: yes. That is a person like you. What are they doing here in Buruburu to prevent  diabetes?  R: I can say it is becaue of eating healthy. That is what I can say. This si becaue there are  times we can be sitting with the boys, and we say, “Such and such a thing are a big  problem, what can we do?” a person will come up with an idea and say that it is about  eating healthy and exercising, mmm taking fruits and avoiding the junk foods.  although as we relax, it eeh reaches a point where we do not put this to action, for  example I can say, “Stop consuming these junky foods, the sugar is not good” I stopped  consuming sugar, I do not consume sugar at all. Eeh, I even see my friends say that they  stopped taking sugar. That is the industrial sugar by the way. People are exercising a lot,  people are really exercising as they are supposed to be doing.  M: okay.  R: yeah.  M: you have said that you sit with the boys and debate on the sugar,  R: mmm  M: where do you meet  R: mmm  M: and how does such a discussion come up?  R: eeh, okay, we do meet, let’s say like tomorrow, mmm and then with tomorrow we will be  at the court, this person will come and the other person will join us. They will say, “Hey,  so and so’s father is having a stroke. So and so’s father is having a stroke. Things are  very bad. With the way the lifestyle is handling us, will we get to that point?”, so, the  discussion will begin like that in the ‘court’.  M: okay. Is it at the basketball’ or where is it?  R: no, the ‘court’, you do not know the ‘court’? With the way the houses in Buruburu are  constructed, we have aah a gate and then there are houses in there. we have the long gate  at the exit, eeh. And now, aah aah, let us say the houses are inside.  M: okay.  R: so, we move within the courts. More especially this time we are having a curfew, people  are more indoors.  M: okay.  R: we also meet at the shopping centre, eeh.  M: okay, so you are saying, there is a sense of community where you know each other  and discuss issues?  R: exactly, that is exactly what frequently happens. So you will find that in Buruburu...do  you know Buruburu?  M: yes, I do, I have passed through, I have been here maybe twice though not so much.  R: eeh, with Buruburu, we have phase 2, I know people from phase 3. You can move from  here and go to phase 3 and find the same-same boys. They do not have to necessarily be  your age mates. They could be even old men, you will find someone’s dad having gone  there and you will chat and say how things are bad. You discuss something, such things.  M: okay.  R: yeah.  M: so you are saying the sense of the community is there because you were brought up  there and know one another?  R: exactly. That is one point, you were brought up there and you know each other.  M: okay.  R: eeh, so, that is all what I can say. People properly know each other.  M: okay  R: yeah  M: would you say that diabetes has stigma?  R: I can’t say that the stigma is there, this is becaue people have started to accept. Eeh, they  have accepted this. People will come and say...they do not hide that they have the  diabetes diseases, ‘so in fact, as we speak, I am supposed to go and buy the medicine’,  eeh, they speak about it and try to live the best way they can. Eeh, it reached a point  where it is no longer shocking. Eeh.  M: okay.  R: yeah. I can give you an example through an experience with a certain woman that I  know of, so, I can greet her and say, “Mom how are you?” She will say, “I want to rush  to the chemist and get the medicine”.  so, as per what I have observed, I think that it is not, people have accepted it. Unlike  long ago when you could be told, “Hey, that guy is HIV positive”, eeh, this is a different  ‘style’. But then even with that, you see currently it is just something...it is not a big deal.  M: yeah.  R: yeah.  M: okay.  R: mmm  M: so do you think because diabetic people are being open about diabetes they are  helping the people with this openness?  R: exactly. You know, if you hear that so and so is diabetic, so, he has been affected by it,  you get shocked. People will wonder what needs to be done becaue it is frequently  happening and the occurrences are too many.  mmm but because you do not have it and you do not know if you are going to get it,  nobody wants to have it, with the people in their 20s, from that age I have told you  around 20 years, they are diabetic. I will say this is becaue of the lifestyle. It has mainly  to do with the lifestyle.  M: okay. Do you think that having a lot of body weight, being overweight is an issue  here in Buruburu?  R: yes, it is there, even young children in class 4, you will find them being very heavy. It is  there. By the way I am not lying about that, it is there. Eeh, you will find that people are  obese. People are obese. When you enquire about it, you will hear that a child ‘hits’  even 6 eggs. Eeh, and this is a person in class 4.  M: I get that.  R: Eeh. So, being overweight is there, it is there.  M: okay.  R: even for old people who are my age, they are trying to work out. If you come to this  place in the morning, you will meet so many people seriously working out.  M: okay.  R: so much. (By madams) {00:16:13} young girls, the men, women, eeh, they work out so  much.  M: do you think the work out is becaue of Corona and the way the people are staying  indoors for long hours or it was there even before corona?  R: it was there before but even right now, eeh, I think corona has contributed to this to some  extent becaue the gyms are also closed. Eeh, so, the people who are there are left  wondering what to do, so they go for jogging in the morning.  M: okay.  R: eeh.  M: in your opinion, what causes a lot of weight in the body?  R: the weight in the body is caused by consumption. Overeating, we eat but we do not think  of the diet, eeh. You know in Buruburu there is a lot of junk food. You understand?  M: mhm.  R: eeh, junk food is there and people also buy the junk food so much. eeh, that also  contributes to that.  M: okay.  R: and then you can find a person having about 5 or 6 eggs in their daily diet, you are not  going to ‘burn’ this during a workout, you are going to grow big. Yeah.  M: there are some people who say that ‘being overweight is god’s wish’,  R: eeh {laughs}  M: what do you think of such an opinion?  R: aah, each person is entitled to his or her own opinion. {Laughs} there is a person who will  think of it that way but according to me, I can’t say that it is a good thing. Eeh, I can’t  say that it is a good thing. You know you will become heavy right now.  for example, a child of 5 years can be too heavy, they will be overweight. Now, by the  time you are clocking 40, if you do not lose something, my friend, I do not think that  things will go well for you. Eeh, if you continue like that and turn obese, then that is how  now things will start affecting you. You will have difficulty in breathing, eeh, just like  we have said diabetes will come in, eeh, and some other things. You see? So, in my  opinion, it is not good. |
| 13 | M: okay. Do you think there is anything that the people of Buruburu are doing to  prevent diabetes in the community?  R: mmm, with the person I can have in mind, yes...or if I find a person with it, if in the  community we are open enough to each other in our households, if my mother, father or  siblings suffer from diabetes, I can help them more with doing exercise, we can walk here  and there and I can try to sensitize to them that yes, eeh, you are supposed to eat things  that are not sugary but I am not sure of the exact foods you are supposed to be eating. If I  am sure about that information, I can be able to assist them more.  M: okay.  R: eeh.  M: that ‘openness’ is not there?  R: most of the times, in this neighborhood, you will find that people stay at their places and  it is not easy to find a person coming out and saying that they have a problem or even  saying that, ‘my father or mother or sibling is suffering from a certain illness.”, you see?  M: mmm.  R: eeh, aah, long ago I had a child that was playing ball games with me. It reached a  particular time and he told me, “it is my time to go and take medication because I am  diabetic.”, when I followed up on this, I was not allowed to go to their home and they  were quite new in the team. As a result of that, I was not able to follow up on that.  M: okay.  R: so, people are no open, leave alone being open, there is no community education, that  education to the public on such issues where people can come out and help one another  on going forward. With HIV, people have come out on HIV saying, “Hey, we have it.  let’s do this and this.” People fear the sensitization and they say, “Hey, no”. so if this  can be brought out by the people you are working on the research with, if you can think  of a way where you can try to bring this near the people so that they can come out and  open up more on this, we can get many people who are sick and they can even test and  get treatment.  M: okay. We can now move to a different topic which is body weight. We will talk of  being overweight.  R: ehe.  M: do you think this is a problem that is in Buruburu and that many people are  affected by this?  R: eeh, with this, I can say that yes it is there. Yes we have people who you will see eating  eeh, foods with a lot of oil in it, you see?  M: mmm.  R: you can also advice the people, eeh, a few of them, you know this also depends on the  age, if you are still young at about 32 years, when they get to a certain point, you can tell  them that it is good to do certain things at that age so that they can avoid issues.  some people will ignore you and think that you are interfering with their lifestyle. You  will find that these people are fat, they do not exercise, they drink eeh alcohol, they feed  on food with a lot of oil and they do not exercise.  M: okay  R: but, that is there. They will not listen to what you are saying although at times you can  meet (an obese person, it could be a woman but then you will not tell them that).  M: okay. You have said that this is because of eating food with a lot of oil and not  exercising,  R: mmm  M: is there anything else that can be the reason as to why people have too much body  weight?  R: weight, eeh, it is there. Alcohol is there, you will find that it makes a person who might  be small bodied to be having a sagging belly, it makes them to have a big stomach.  When a person is obese they will have breathing problems. You see?  M: mmm.  R: if their body is flexible and they can be able to do exercise here and there, you will find  that they will not have issues, eeh, the heart will properly pump the blood. {Loud background  sweeping sound} yeah, with foods like alcohol, foods with a lot of oil or a lot of sugar, we  would be very happy if people fed on the right foods.  M: okay. There are people who do say that being heavy weight or fat is god’s wish.  What is your opinion on that?  R: {laughs} I do not accept that. {Laughs} even if it is God’s wish we can get to control things  and if the body is big you control it. When you eat, sugar and sugary foods will make  your body to have more weight. You see? |
| 14 | M: so is the level of awareness sufficient? Do people know the harmful effects of  weight? Do they know how they could lose weight?  R: I think people would be knowing, aah, how to do weight but, but the real information is  not there. People might be having information that is sketchy, sketchy information, I  think intensified awareness could. So that you know you have, you have the real, the real  facts, you have the real knowledge, how to go about the issues affecting people.  M: okay.  R: you could be knowing maybe one aspect. “Okay, let me do exercise to lose weight”.  Maybe again, how do you do that exercise? Somebody may not be knowing.  M: okay.  R: I need, I need not to eat food, I need to check on my diet. But what kind of food do I  need not to eat. I think that, that, that in-depth knowledge, that in-depth awareness is  what is lacking. So, people have a skewed or sketchy information on what to do.  M: okay.  R: yes.  M: what about the issue of the community perception? You mentioned that if you lose  weight, people will start thinking that you are sick or you have a problem,  R: mmm  M: will that prevent someone from losing weight and how can we rectify that?  R: and I think just what we said, through awareness.  M: yes.  R: because by that, people will be able to know that if people lose weight they are not losing  weight because they are sic, yeah he is not losing weight becaue he has contacted  HIV/AIDs, he is not losing weight because things have become bad economically for  him. Because the moment you are losing weight, like the perception is, “Nowadays so  and so has a lot of problems.” When you are gaining weight, “Oh, so and so is doing  well.” Yeah?  M: yes.  R: so, those kinds of misconceptions. I think with awareness, people need to know that even  if you are gaining or losing weight, it is not a fact that you are not doing well  economically. You are trying to check on your physical life.  M: okay.  R: yeah.  M: is there anything else that can be done to help people lose weight in Buruburu apart  from awareness, you also mentioned discipline,  R: yes,  M: what else can help people to lose weight?  R: mmm maybe let’s say if having apart from the issues of, of awareness and the, and the,  and the, and those issues of that discipline, yeah. just, maybe trainings, workshops and  maybe people can determine like local NGOs around, they can come up with the  resources and places where people can b doing exercises, maybe the gyms and such  things of the sort, I think it can be good.  M: okay.  R: especially like that age group that we had said,  M: yes,  R: people can come up with the, with places where they can all be having the leisure time,  so, they don’t just sit in the house, sit and watch TV, as in a place whereby even his or  her leisure time, they can be able to do something, things like recreational centers.  M: yes.  R: yes.  M: as of now in Buruburu, do you have any recreation centers, do you have gyms,  R: the gyms are there but some people are able to afford, some people are not able to afford.  But I haven’t come across a recreation, a youth recreational centre whereby maybe that  particular age group is able just to go sit there and maybe dedicate the time. Maybe come  and do arts, come and do sports, to come and do eeh, things of the sort. There are some  things that will just dedicate them physically. Yeah.  M: okay.  R: yes.  M: we move to something else which is on diet.  R: yes.  M: which are the most common foods that you take in your household?  R: mmm, me, like me I take a lot of starch, aah I take ugali on a daily basis.  M: yes.  R: yeah. So, maybe I will be mixing it with some proteins, and basically with some  (vegetables).  M: okay.  R: yeah.  M: and why do you take these foods?  R: mmm, I trust that when I take ugali, then I am good. I will feel like I am satisfied, I am  able to have the right calories in my body. Yeah.  M: okay, apart from that anything else?  R: mmm, in terms of food? No. |
| 15 | M: how do people perceive physical activity, is it something that is accepted, ignored?  R: aam, well?  M: in Buruburu.  R: 50, 50/50. There are those ones who like it. Like you see, with our government, during  this time of the pandemic we have been told to stay at home, have you ever realized that  so many people were, were going for jogging?  M: mhm.  R: quite a lot of people. People staying in one court can say that they will be going for  jogging in the morning. That shows, people like physical whatever, they go for jogging  in the whole of Buruburu, go through (ACK) {00:25:38} and come back through Uchumi.  Yeah and it was quite a big group that so many people joined in.  M: okay.  R: so to me that was very encouraging. So that is the kind of good exercise. Mmm. Yeah.  M: do you think this change will be sustainable after Corona?  R: I am thinking it will because people will have already adapted. Yes, I am seeing, I, I,  think of it continuing,  M: okay  R: people have already adapted and you see they have adapted becaue of this issue of just  staying at home and eating and sitting you are thinking you are adding weight, so, it has  affected many people. It has become a norm for most people and I think it is going to  continue.  M: okay.  R: mmm.  M: you talked of walking,  R: mmm  M: you walk for your physical activity,  R: yes.  M: how is walking perceived, if someone saw you walking from Buruburu to Donholm,  R: mmm  M: won’t they think you do not have fare or things like that?  R: yeah now there is a problem if that is what people think but as long as you know the  intentions you have in your own mind, people will, people are there to talk, and the  society, society will always say, the society will say so many things about you but for me  that, that doesn’t bother me much.  M: okay.  R: yeah, I really don’t care what you think.  M: okay.  R: but for me I know for my age that is very healthy for me. So whatever they feel now, is  up to them, but for me I know it is helping me in a way and it is healthy for me. |
