# Supplementary figures and images for: Perceptions of diabetes risk and prevention in Nairobi, Kenya: A qualitative and theory of change development study
Source: PLoS One. 2024 Feb 13;19(2):e0297779. doi: 10.1371/journal.pone.0297779 (PMC10863861; doi:10.1371/journal.pone.0297779)

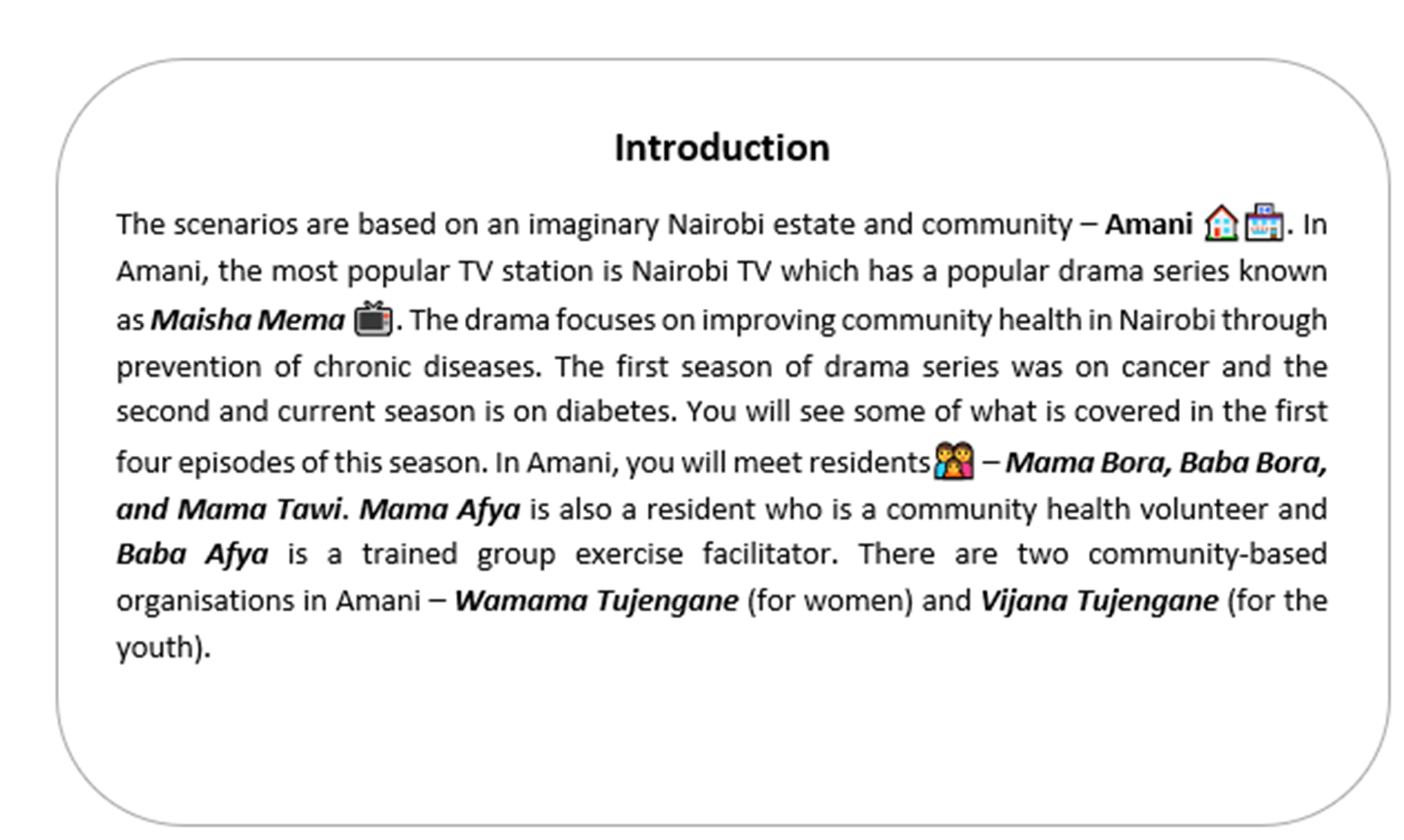

Supplement: S1 Fig — (ZIP) [file pone.0297779.s004.zip › S1 Fig 1.png]

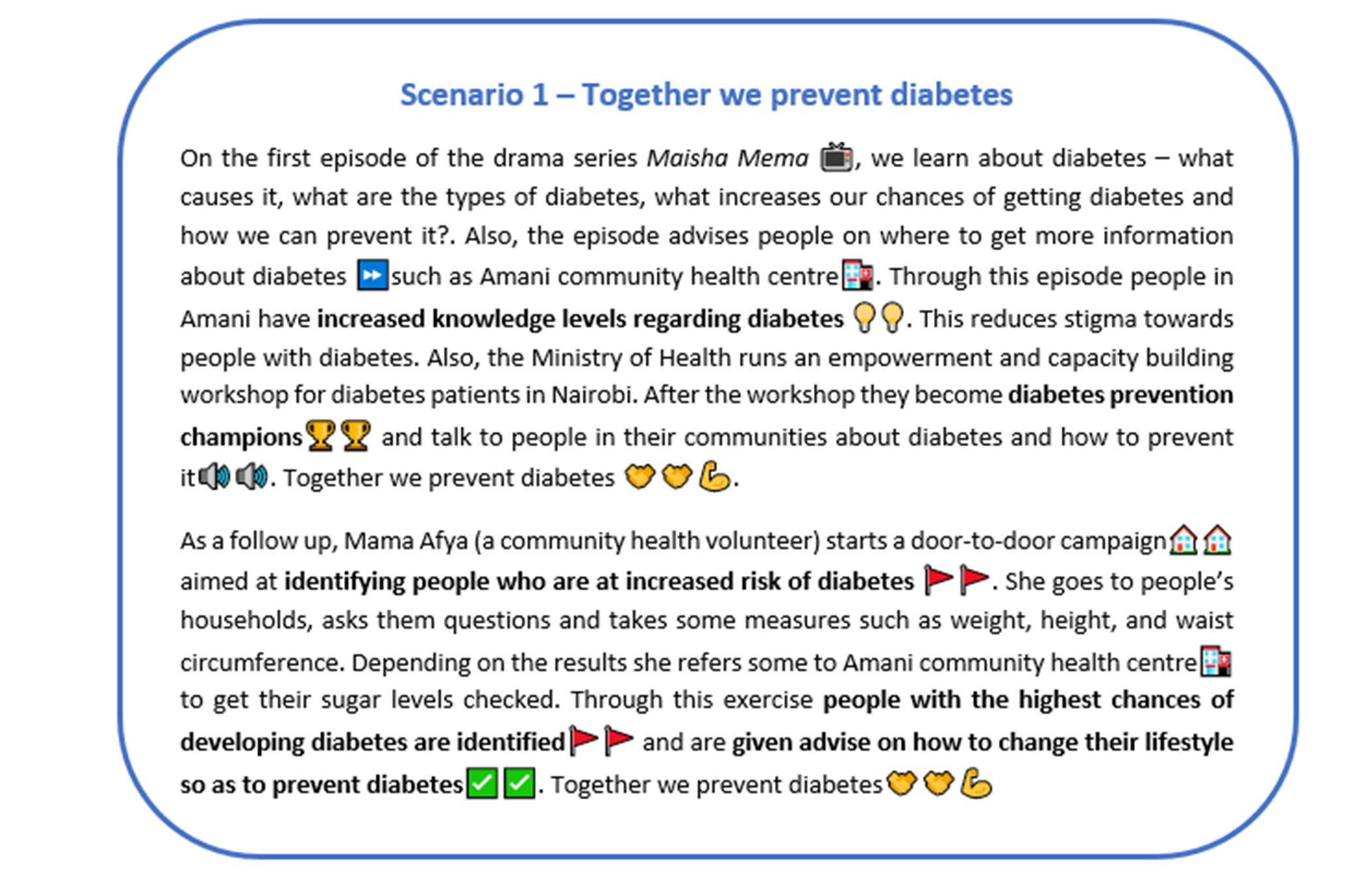

Supplement: S1 Fig — (ZIP) [file pone.0297779.s004.zip › S1 Fig 2.png]

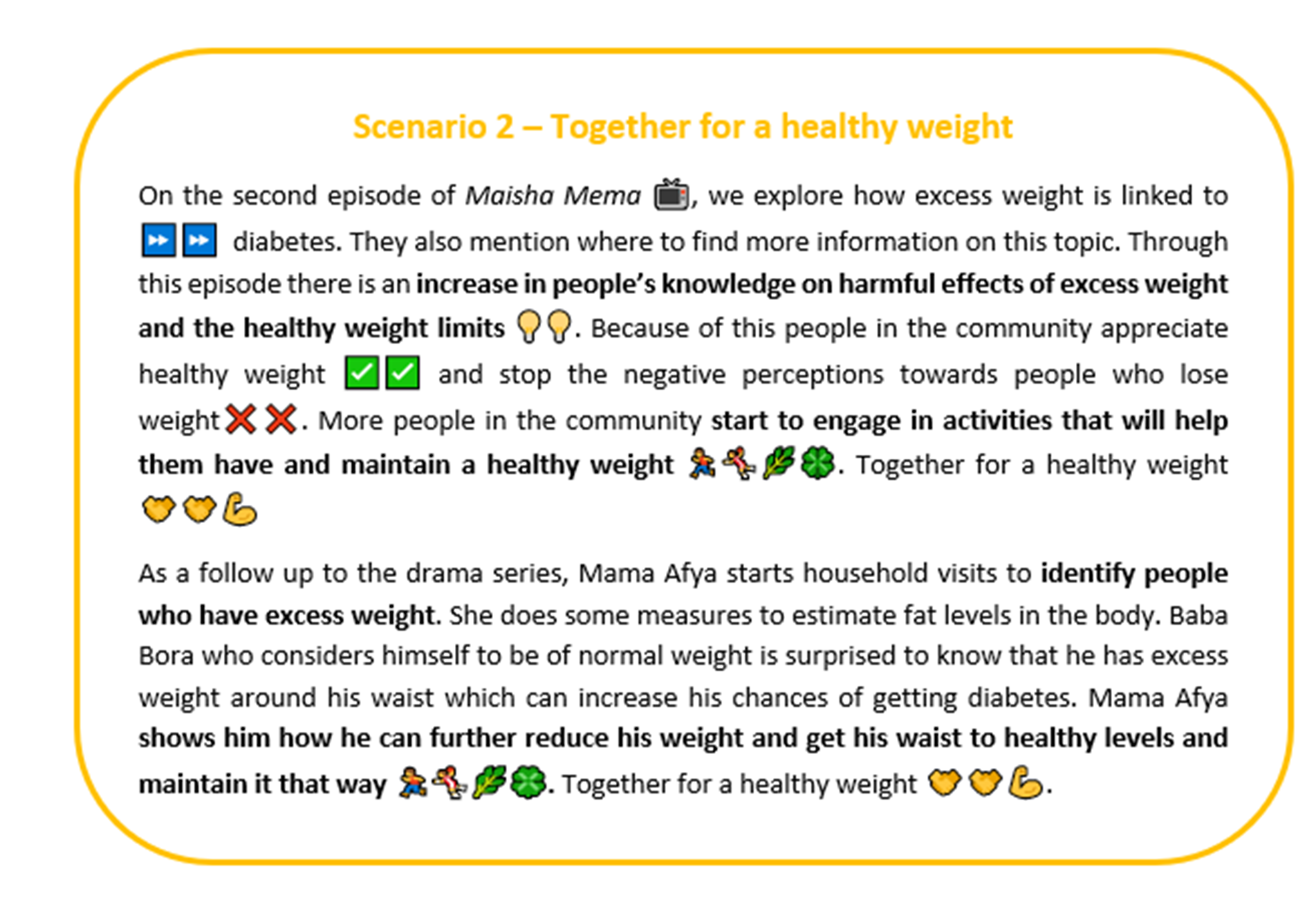

Supplement: S1 Fig — (ZIP) [file pone.0297779.s004.zip › S1 Fig 3.png]

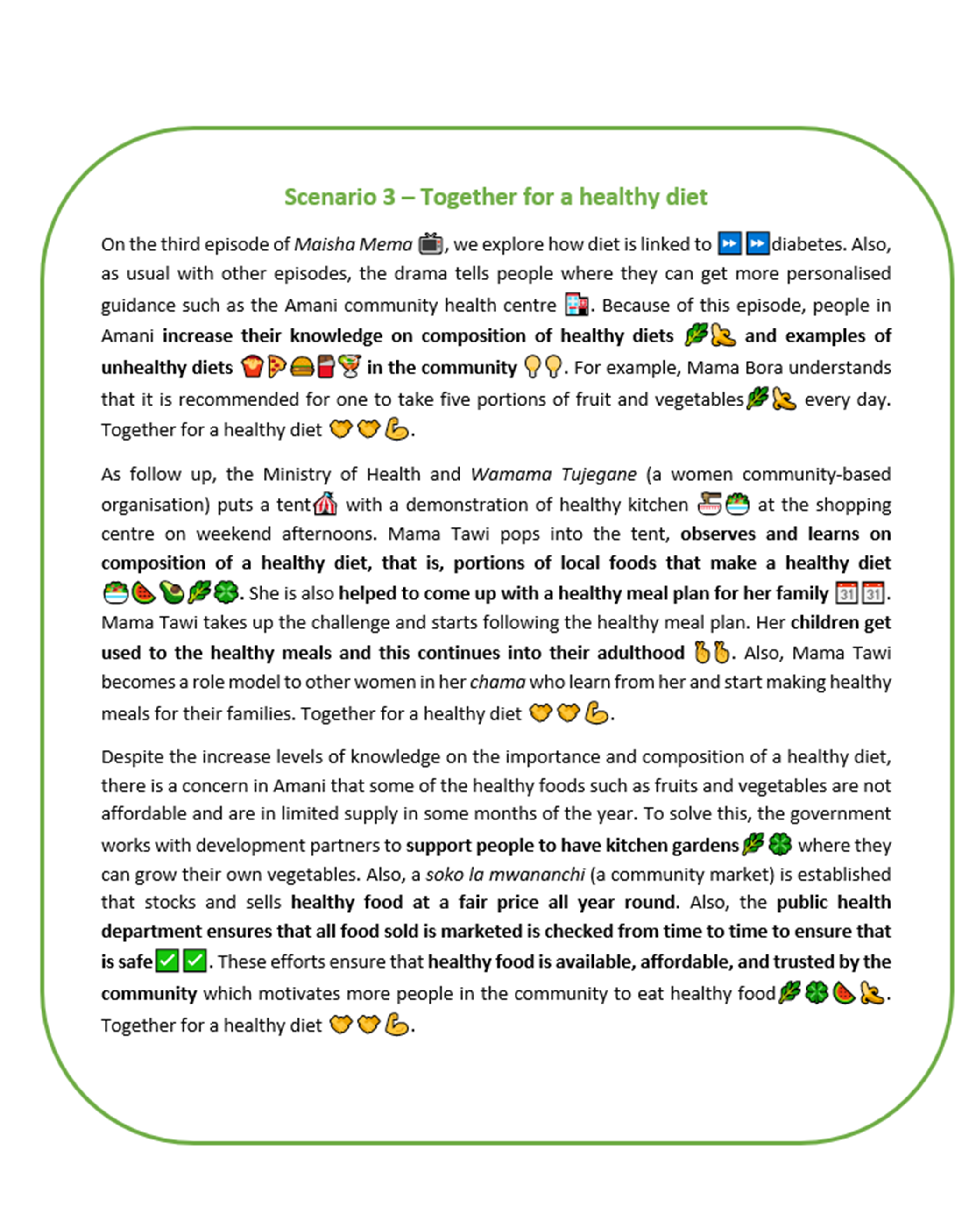

Supplement: S1 Fig — (ZIP) [file pone.0297779.s004.zip › S1 Fig 4.png]

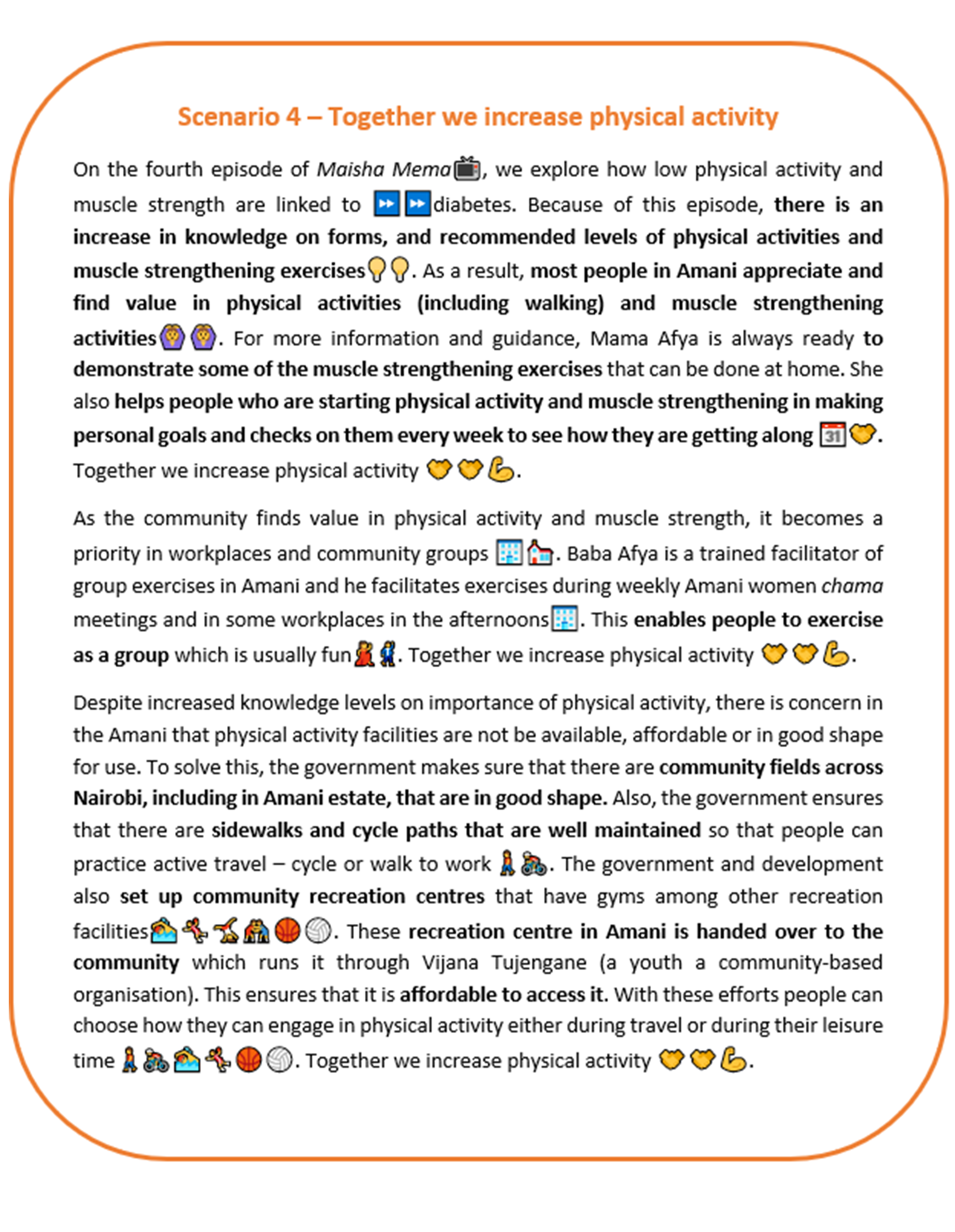

Supplement: S1 Fig — (ZIP) [file pone.0297779.s004.zip › S1 Fig 5.png]

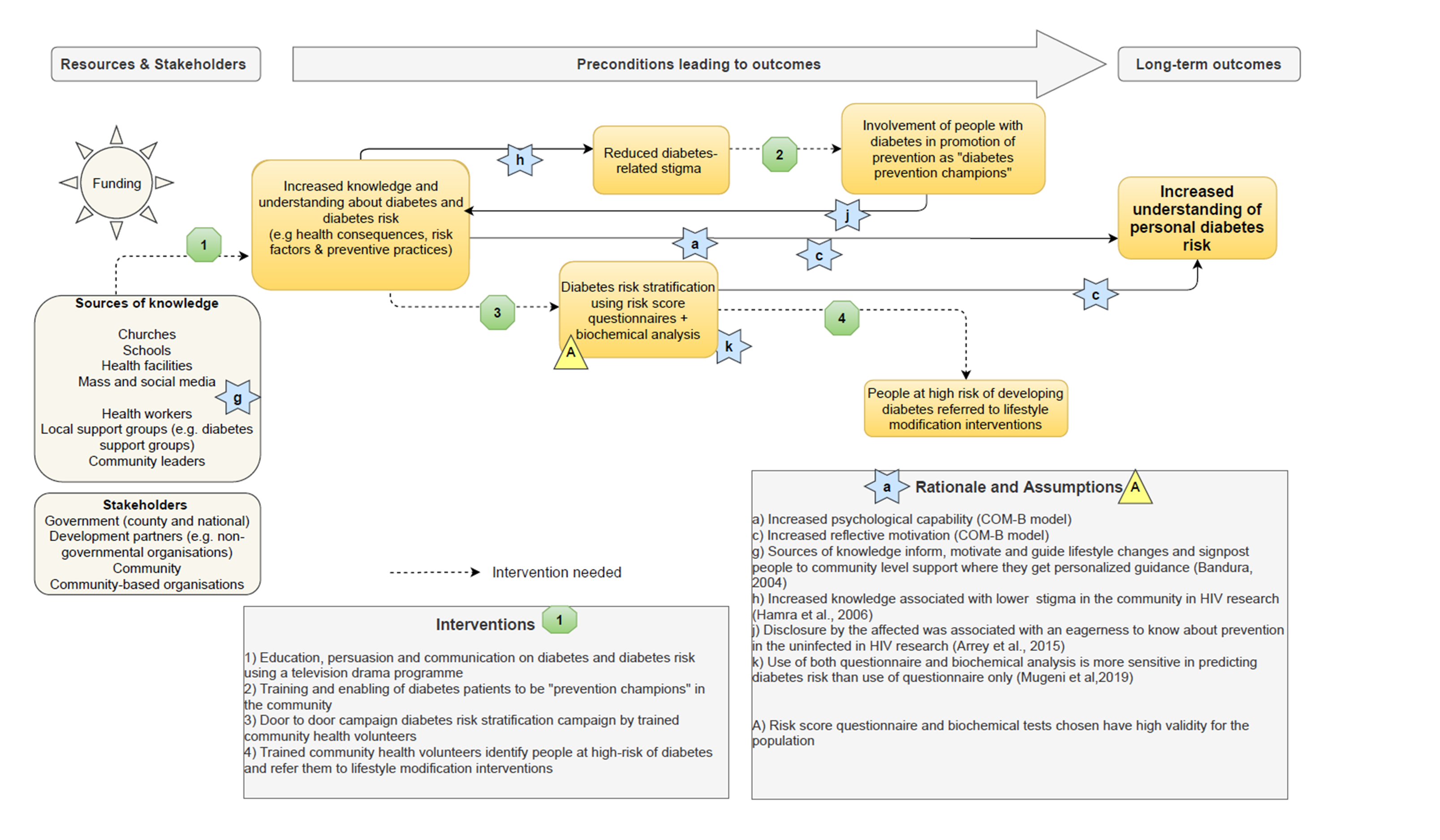

Supplement: S1 Fig — (ZIP) [file pone.0297779.s004.zip › S1 Fig 6.png]

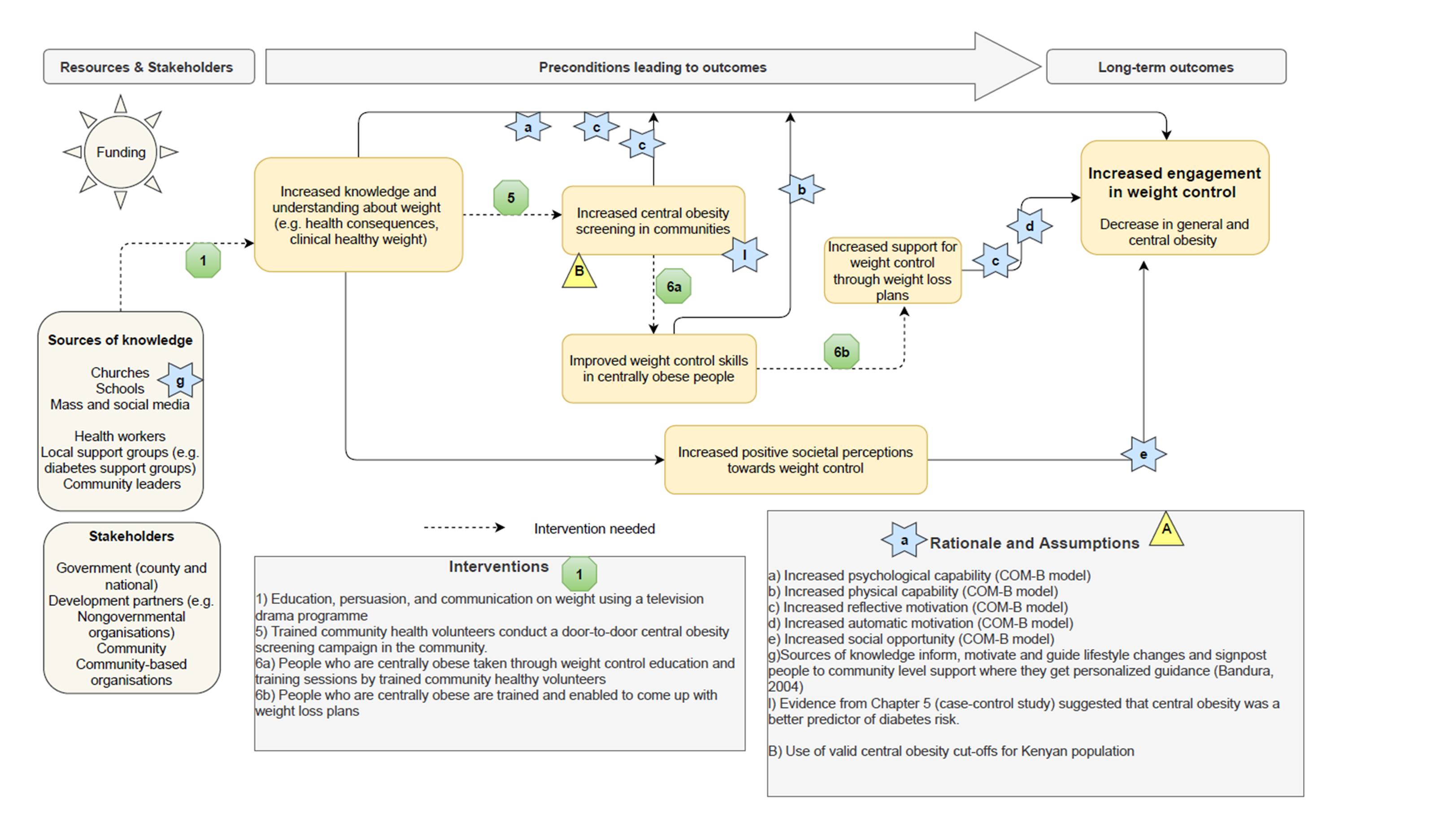

Supplement: S1 Fig — (ZIP) [file pone.0297779.s004.zip › S1 Fig 7.png]

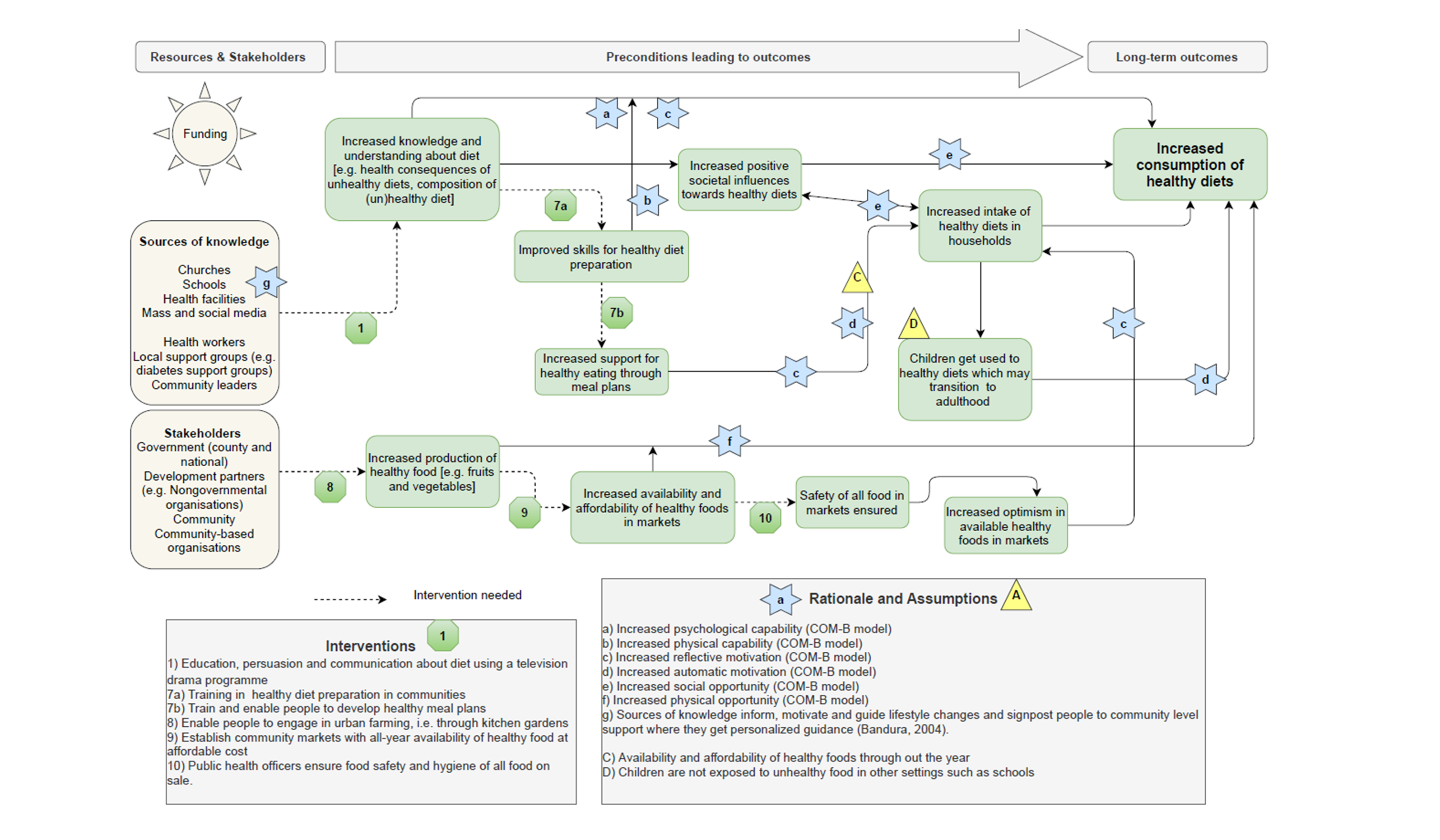

Supplement: S1 Fig — (ZIP) [file pone.0297779.s004.zip › S1 Fig 8.png]

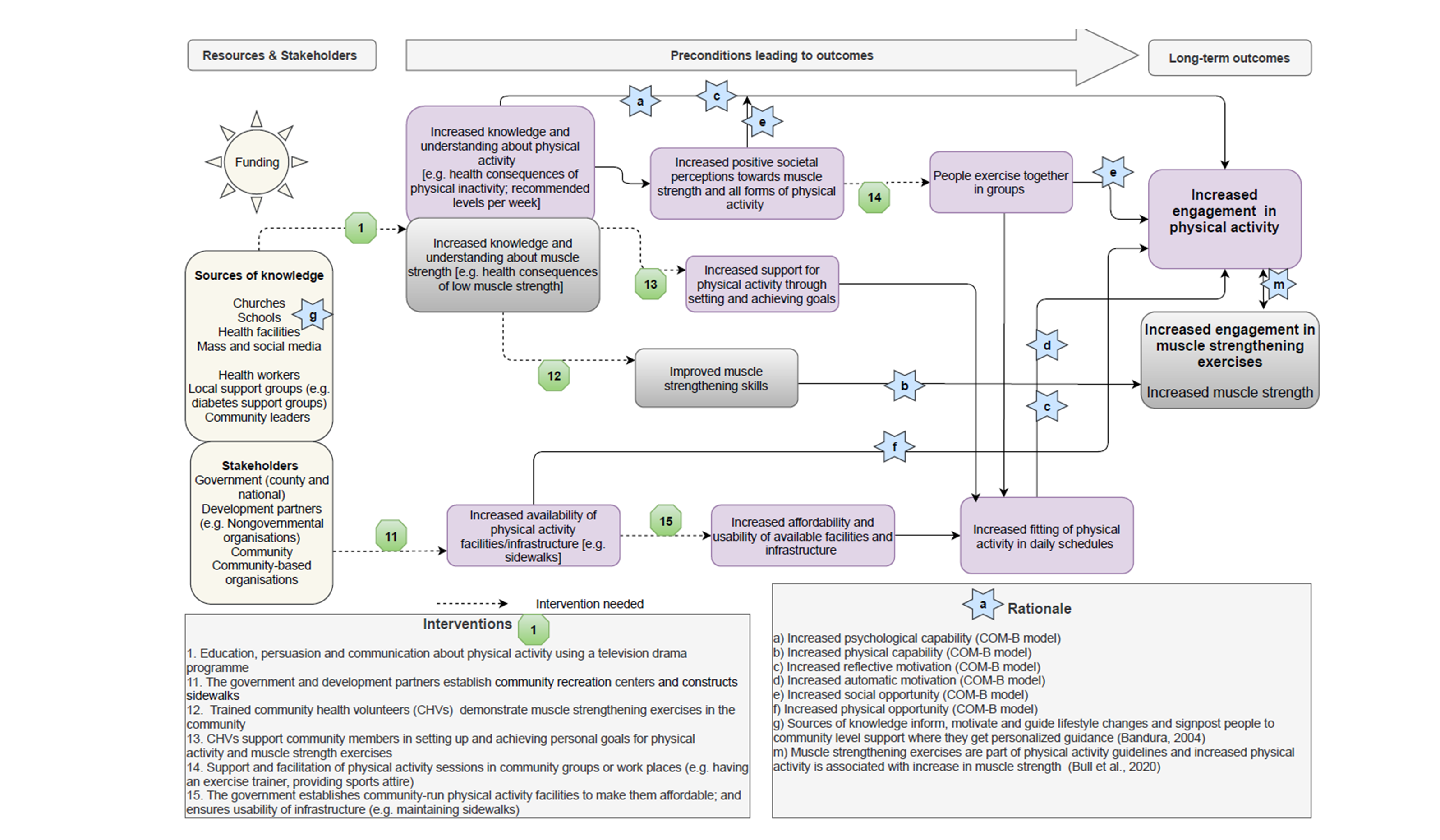

Supplement: S1 Fig — (ZIP) [file pone.0297779.s004.zip › S1 Fig 9.png]
